# Predictive capacity of paediatric nasal epithelial cells in sequential CFTR modulator therapy

**DOI:** 10.1136/thorax-2025-223153

**Published:** 2025-12-07

**Authors:** Laura Katherine Fawcett, Zahra A Chew, Elena K Schneider-Futschik, Katelin M Allan, Hardip R Patel, Adam Jaffe, Shafagh Waters

**Affiliations:** 1 Discipline of Paediatrics and Child health, School of Clinical Medicine, Faculty of Medicine and Health, UNSW Sydney, Sydney, New South Wales, Australia; 2 Department of Respiratory Medicine, Sydney Children’s Hospital Randwick, Sydney, New South Wales, Australia; 3 John Curtin School of Medical Research, Australian National University, Canberra, Australian Capital Territory, Australia; 4 School of Biomedical Sciences, Faculty of Medicine and Health, UNSW Sydney, Sydney, New South Wales, Australia; 5 Department of Biochemistry and Pharmacology, School of Biomedical Sciences, Faculty of Medicine, Dentistry and Health Sciences, The University of Melbourne, Melbourne, Victoria, Australia

**Keywords:** Cystic Fibrosis, Airway Epithelium

## Abstract

**Background:**

CFTR modulators have transformed cystic fibrosis (CF) treatment, but individual responses vary even among patients with identical *CFTR* genotypes. This underscores the need for predictive biomarkers to optimise therapeutic selection.

**Methods:**

We evaluated 24 paediatric patients homozygous for F508del-*CFTR*, assessing lung function (FEV1pp) and sweat chloride (SC) before and after CFTR modulator therapy. Whole-gene sequencing was utilised to identify *CFTR* and pharmacogene variants. Patient-derived human nasal epithelial cells (HNECs) were expanded and differentiated at the air-liquid interface to assess CFTR function via ion transport (ΔIsc).

**Results:**

Clinical responses varied widely. Twelve participants changed modulators during the study. Sequencing identified 231 additional *CFTR* variants and pharmacogene polymorphisms, but none correlated with response variability. However, a significant linear relationship emerged between ΔIsc and FEV1pp improvement in patients with baseline FEV1pp<90 (R² = 0.652, p =0.001) and SC reduction (R² = 0.535, p=0.004). Receiver operating characteristic analysis demonstrated high predictive accuracy for SC reduction (area under the curve (AUC)=0.88) and combined FEV1pp/SC response in patients with baseline FEV1pp<90 (AUC=1.00). Exploratory analysis confirmed that ΔIsc predicts FEV1pp changes, modulated by baseline lung function and CFTR modulator type.

**Conclusion:**

Patient-derived differentiated HNEC cultures serve as a robust predictive tool for CFTR modulator response in paediatric CF patients. Their integration into clinical practice can enhance personalised treatment strategies, minimising ineffective therapy use and improving CF patient outcomes with precision medicine.

**WHAT IS ALREADY KNOWN ON THIS TOPIC**

- CFTR modulators significantly improve clinical outcomes in people with cystic fibrosis (CF), yet individual responses vary, even among those with identical *CFTR* genotypes.
- Patient-derived human nasal epithelial cell (HNEC) models have emerged as promising tools to predict CFTR modulator response. However, existing studies have primarily focused on adult and adolescent populations, leaving a gap in personalised treatment strategies for younger children with CF.
- The relationship between *CFTR* sequence variations, pharmacogene heterogeneity and modulator response in paediatric patients has not been extensively explored.

**WHAT THIS STUDY ADDS**

- This study demonstrates a strong correlation between in vitro CFTR function (ΔIsc) and clinical improvements in FEV1pp and sweat chloride in children and adolescents with CF.
- Whole-gene sequencing identified 231 additional CFTR variants, yet none were associated with CFTR modulator response, suggesting that genotype alone does not fully explain treatment variability.
- While some trends between pharmacogene activity and treatment response were observed, no strong evidence supports pharmacogene profiling as a standalone predictor of CFTR modulator efficacy in paediatric patients.
- Differentiated-HNEC cultures consistently predicted clinical response across multiple CFTR modulator regimens, reinforcing their value for preclinical drug screening.

**HOW THIS STUDY MIGHT AFFECT RESEARCH, PRACTICE OR POLICY**

- Our findings support the integration of differentiated-HNEC models into clinical practice to personalise CFTR modulator selection, reducing ineffective treatments and improving patient outcomes.
- The study underscores the need for additional clinical endpoints beyond FEV1pp to assess respiratory function in individuals with preserved lung function (FEV1pp>90%).

## Introduction

 Cystic fibrosis (CF) is an autosomal recessive disorder caused by pathological variants in the CF transmembrane conductance regulator (*CFTR*) gene, that encodes a chloride and bicarbonate channel.^1^ The most prevalent CF-causing variant, F508del, results in CFTR protein misfolding, premature degradation and impaired gating function.^2^ The development of CFTR modulators, such as potentiators like ivacaftor (IVA; I) and correctors such as lumacaftor (LUM), tezacaftor (TEZ; T) and elexacaftor (E), has revolutionised treatment for people with CF (PwCF). These modulators, when used as dual or triple therapy, target specific CFTR variants like F508del, to address the multifactorial dysfunction caused by this variant.^3^

Implementation of CFTR modulators to the clinic faces challenges due to the considerable heterogeneity in clinical responses between PwCF.^4^,^6^ Current clinical tools to assess response to CFTR modulator therapy include measurement of forced expiratory volume in one second (FEV1) and sweat chloride (SC) levels. FEV1 has been the primary endpoint in most phase III trials; however, its limitations, such as reduced sensitivity to early lung disease changes, have led to exclusion of individuals with FEV1 values above 90 per cent predicted (pp).^6 7^ SC, the diagnostic gold standard, is now also used as a biomarker for CFTR modulator efficacy.^8^ Changes in clinical endpoints are continuous; however, an increase in FEV1pp by more than 5 percentage points and reduction in SC by more than 20 mmol/L have typically been accepted to indicate a significant positive therapeutic response to treatment.^8^,^11^

Accurately predicting the clinical response is important because incorrect or suboptimal treatments may lead to increased healthcare costs and reduced quality of life due to adverse side effects.^1^ When variability in CFTR modulator efficacy between PwCF is not due to their adherence to treatment, it may be explained by drug-drug interactions with concomitant CF therapies.^12 13^ Alternatively, genetic modifiers such as polymorphisms in drug metabolising enzymes, pharmacogenes, can result in poor metabolisers or ultra-rapid metabolisers of CFTR modulators.^13 14^ Complex *CFTR* alleles, additional variants present with F508del, have also been shown to impact clinical response to CFTR modulators.^15 16^ Standard clinical *CFTR* genotyping may overlook these additional variants, highlighting the importance of comprehensive genetic profiling to accurately identify responders.

To address the challenge of predicting individual responses to CFTR modulators, preclinical methodologies using patient-derived primary cell models have emerged as promising tools, which are also acceptable to patients.^17^,^19^ These models, derived from the airway (nasal, bronchial) and intestinal epithelial cells, enable characterisation of the molecular and functional defects caused by *CFTR* variants in a biologically relevant system, as well as assessment of a therapy’s efficacy in modulating CFTR protein activity.^20 21^ Differentiation of human airway cells into pseudostratified epithelium at air-liquid interface (ALI) facilitates direct measurement of ion transport, enabling accurate evaluation of CFTR function and drug efficacy.^22^,^24^ Differentiated human nasal epithelial cells (HNECs), validated for CF modulator drug testing, offer non-invasive accessibility compared with bronchial cells.^25^ The gold standard for evaluating CFTR function involves electrophysiological ion transport assessments, specifically measuring short-circuit current (Isc) in the Ussing chamber to detect changes in cAMP-mediated chloride (Cl^-^) transport induced by CFTR modulators.^26 27^

Several studies have demonstrated a strong positive correlation between ΔIsc measurements in paired differentiated-HNEC cultures and the clinical response to CFTR modulators, in both common and rare *CFTR* variants (online supplemental table S1).^2428^,^31^ Pranke and Debley found a strong correlation between ΔIsc in differentiated-HNEC cultures and changes in FEV1pp and SC in PwCF with F508del-*CFTR* and gating variants treated with LUM/IVA and IVA, respectively.^24 29 30^ Dreano reported similar correlations in a diverse cohort of non F508del-*CFTR* PwCF.^28^ Additional studies have provided individual results for those with rare CF-causing *CFTR* variants, demonstrating the broader applicability of these findings.^32^,^34^

Although insightful, these studies focus primarily on adult and adolescent participants. Children with CF represent prime candidates for personalised treatment selection due to their potential for long-term benefit if effective therapy is initiated early. Early modulator use has been associated with restoration of pancreatic function in infants, and initiation prior to irreversible airway damage may offer lifelong benefits. However, predicting response remains difficult due to challenges in clinical monitoring in this population. Functional testing in patient-derived cells may enable modulator selection in young children with minimal disease burden, where conventional endpoints are limited.

**Figure 1:**
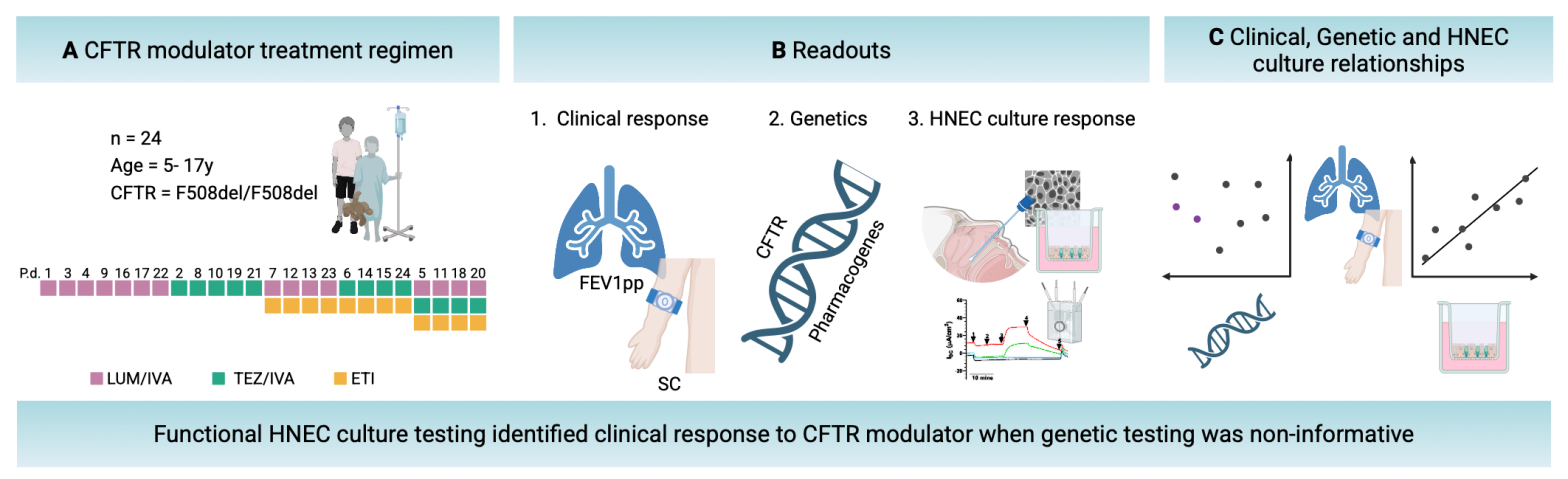
Overview of study design and analytical framework. (A) CFTR modulator treatment regimen. Each box on the first row represents one of the 24 study participants (F508del/F508del CFTR, Aged 5–17 years) P.d; participant de-identifier. Subsequent rows illustrate changes in treatment regimen, denoted by the switch in colour within columns. The colour indicates the specific CFTR modulators administered, including LUM/IVA (lumacaftor/ivacaftor), TEZ/IVA (tezacaftor/ivacaftor) or ETI (elexacaftor/tezacaftor/ivacaftor). (B) Readouts. *Clinical response* (in vivo): measured via lung function (FEV1pp) and sweat chloride (SC) pre- and post-treatment. *Genetics:* comprehensive CFTR and pharmacogene sequencing. *Human nasal epithelial cell (HNEC) culture response (in vitro):* assessed using electrophysiological measurements of CFTR function in differentiated-HNEC cultures derived from each participant. (C) Clinical, genetic and HNEC culture relationships. Clinical outcomes, including FEV1 percent predicted and SC levels, are compared with genetic sequencing data and to differentiated-HNEC culture responses to CFTR modulators to evaluate the predictive value of epithelial cell models. FEV1pp: forced expiratory volume in 1 s, percent predicted. Created in BioRender.^43^

In this study, the relationships between in vivo clinical response (FEV1pp and SC), genetics (*CFTR* and pharmacogenes) and in vitro functional response (ΔIsc in differentiated-HNEC) were investigated in PwCF aged under 18 years (figure 1).

## Materials and methods

Detailed materials and methods are available as online supplemental materials.

### Participants

This study was approved by the Sydney children’s Hospital Network (SCHN) Ethics Review Board (HREC/16/SCHN/120). Written informed consent was obtained from all participating subjects and/or their legal guardians. Participants were recruited from the Sydney Children’s Hospital (SCH) CF clinic if they: (1) had an F508del homozygous *CFTR* genotype, (2) commenced treatment with a CFTR modulator with ongoing follow-up by the CF team and (3) completed spirometry testing pre and post modulator treatment according to the American Thoracic/European Respiratory Society criteria.^35^

### In vivo clinical response to modulator: FEV1 pp and SC

Change in FEV1pp was calculated as the difference between baseline FEV1pp and FEV1pp measured after at least 6 weeks of stable CFTR modulator dosing. Due to the progressive nature of CF lung disease, when participants changed CFTR modulator treatment, the FEV1pp measurement obtained immediately prior to the first dose of the subsequent CFTR modulator was used as their subsequent baseline (online supplemental figure M1).

SC testing was performed as part of clinical care of patients receiving CFTR modulators at the SCH CF clinic, and results were obtained from the medical record. Change in SC was the difference between SC levels prior to starting any CFTR modulator treatment and after at least 4 weeks of stable CFTR modulator dosing. When a SC level was not available immediately prior to commencing CFTR modulator treatment, any prior result was used as baseline (n=2 comparisons).

### Creation of in vitro nasal epithelial cell cultures

Passage 1 cryopreserved HNECs were seeded (200–250 000/filter) onto collagen-I coated 6.5 mm Transwell membrane inserts (Sigma CLS3470) as previously described.^36^ These HNECs were expanded using PneumaCult Ex Plus media (STEMCELL Technologies 05040) until confluent (4–6 days). The basal media was replaced with ALI differentiation media (STEMCELL Technologies 05001), and an ALI was created by removal of the apical media. The HNECs were maintained at ALI for 21 days with alternate basal media changes before CFTR-mediated ion transport was assessed by measurement of short circuit current (I_SC_) in the differentiated-HNEC cultures as described previously (online supplemental file 2).^27^

### Statistical analysis

Prior to enrolment, power calculations were performed for the primary outcome (relationship between first CFTR modulator and change in FEV1pp). Assuming a correlation of 0.6 and sample size of 25, the 95% CI width for the regression slope is no greater than 1.25.

Change in clinical measurements of FEV1pp and SC was evaluated using a paired t test. Waterfall plots were used to visualise the variations in clinical response between individuals. Participants were stratified into two groups for analysis based on their baseline FEV1pp; group 1, FEV1pp 40–90; and group 2, FEV1pp<40 or > 90, although no participants had an FEV1pp less than 40 at baseline. Participants did not undergo a washout period before changing modulator treatment, so subsequent baseline values are confounded by prior therapy. Therefore, the relationship between clinical and in vitro functional response (ΔIsc) to only the first CFTR modulator was evaluated using multiple regression analysis, which included age, gender and an interaction with baseline FEV1pp. Receiver operating characteristic (ROC) curves were generated for in vitro functional response (ΔIsc) that identified predefined significant clinical response. A significant increase in FEV1pp was considered an increase in absolute pp greater than 5 percentage points. A significant improvement in SC was considered a decrease of greater than 20 mmol/L.

To investigate the full data set available, which included responses to subsequent modulator treatment, we used generalised estimating equations with an unstructured covariance matrix to account for repeated measures. The model for change in FEV1pp included fixed effects for age, gender, CFTR modulator, baseline FEV1pp group, in vitro functional response (ΔIsc) and an interaction between baseline FEV1pp group and in vitro functional response (ΔIsc). The model for change in SC did not include baseline FEV1pp group. Residuals were checked, and sensitivity analysis was run when model assumptions were not met by the data. Statistical analysis was performed with GraphPad Prism software (v9.5.1) and R (v4.2.2). A *p* value of less than 0.05 was considered statistically significant.

## Results

### Baseline characteristics and modulator treatment regimen

In this study, 24 PwCF aged between 5 and 17 years, all homozygous for the F508del-*CFTR* variant met the inclusion criteria (table 1, online supplemental table S2). Prior to treatment with modulator therapy (baseline), every participant exhibited an FEV1pp above 40, with half of the group having an FEV1pp above 90 (table 1, online supplemental table S2). Each participant received an age-appropriate CFTR modulator as part of their comprehensive CF management, with 15 participants receiving LUM/IVA and nine participants receiving TEZ/IVA. Twelve participants underwent changes in their CFTR modulator regimen during the study period (figure 1A). Measurement of CFTR modulator levels in participants’ blood samples confirmed adherence to the prescribed regimen, with median LUM detected 48.35 µg/mL and median TEZ detected 47.88 µg/mL (online supplemental figure S1). Data on CFTR modulator dose timing in relation to sample acquisition were not recorded in the participants’ medical record and therefore unavailable to us.

**Table 1:** Participant characteristics

Baseline FEV1pp	40–120	< 90	≥90
n	24	12	12
Female	18 (75%)	10 (83%)	8 (66.67%)
Age (years)	12.61 (3.38)	15.68 (1.16)	11.12 (3.04)
Age groups (years)			
5–11	9 (37.5%)	3 (25%)	6 (50%)
12–17	15 (62.5%)	9 (75%)	6 (50%)
FEV1pp	85.39 (19.29)	69.17 (13.10)	101.6 (5.74)
BMI (CDC percentile*)	51.64 (28.89)	44.1 (27.22)	59.17 (29.67)
SC (mmol/L)	97 (11.16) (n=19)	99.5 (3.07) (n=8)	95.6 (14.52) (n=11)
First modulator treatment			
LUM/IVA	15 (62.5%)	7 (58.33%)	8 (66.67%)
TEZ/IVA	9 (37.5%)	5 (41.67%)	4 (33.33%)
Switched to TEZ/IVA†	4 (16.6%)	2 (16.6%)	2 (16.6%)
Switched to ETI	12 (50%)	6 (50%)	6 (50%)

All participants are F508del homozygous.

n(%) or mean(SD)

* CDC growth charts are routinely used in Australia for children aged over 2 years. SC was collected during routine visits. Missing data occurred when testing was not undertaken as part of routine clinical care.

† All four participants underwent sequential treatment with all three modulators.

BMI, body mass index; CDC, Centres for Disease Control and Prevention; E, elexacaftor; ETI, elexacaftor/tezacaftor/ivacaftor; FEV1pp, forced expiratory volume in 1 s, percent predicted; IVA, ivacaftor; LUM, lumacaftor; SC, sweat chloride; TEZ, tezacaftor.

### Heterogenous in vivo clinical response (FEV1pp and SC)

**Figure 2:**
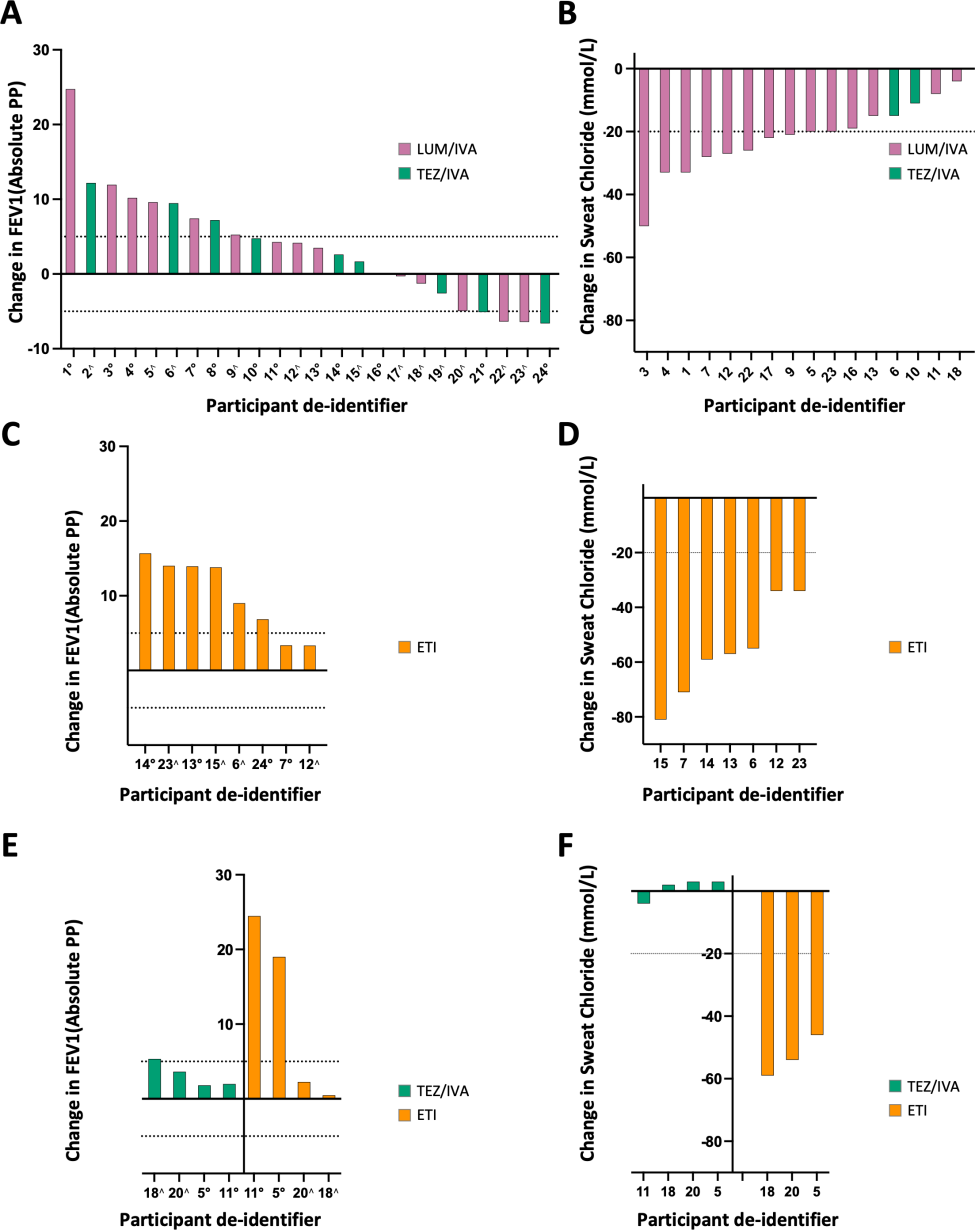
Heterogeneous in vivo clinical response to CFTR modulators. Waterfall plots illustrate the absolute change in participant outcomes after treatment with CFTR modulators. (**A**) Change in FEV1pp. Absolute change in FEV1pp following treatment with lumacaftor/ivacaftor (LUM/IVA) or tezacaftor/ivacaftor (TEZ/IVA). Each bar represents an individual participant, and dotted lines indicate significant changes from baseline. (**B**) Change in SC: absolute change in SC levels after treatment with LUM/IVA or TEZ/IVA. Each bar represents a participant, with significant changes indicated by dotted lines. (**C**) Subsequent change in FEV1: absolute change in FEV1pp after switching directly to elexacaftor/tezacaftor/ivacaftor (ETI). Change is calculated as the difference between the subsequent baseline following treatment with a modulator, taken immediately prior to the new modulator and post-treatment values for the new regimen. (**D**) Subsequent change in SC: Absolute change in SC levels after switching directly to ETI, calculated as the difference between baseline (no treatment) and post-treatment values for the new regimen. (**E**) Subsequent change in FEV1 in participants who transitioned from LUM/IVA to TEZ/IVA before switching to ETI: absolute change in FEV1pp after switching to a subsequent CFTR modulator TEZ/IVA or ETI. Change is calculated as the difference between the subsequent baseline following treatment with a modulator, taken immediately prior to the new modulator, and post treatment values for the new regimen. (F) Subsequent change in SC in participants who transitioned from LUM/IVA to TEZ/IVA before switching to ETI: absolute change in SC levels after switching to TEZ/IVA or ETI, calculated as the difference between baseline (no treatment) and post-treatment values for the new regimen. Participants are identified consistently across all four graphs using a participant deidentifier. Nine participants had incomplete SC data (online supplemental table S2). Symbols denote participants with a baseline FEV1pp below 90 (∘) or above 90 (∧). FEV1pp, forced expiratory volume in 1 s, percent predicted; SC, sweat chloride.

The clinical response of all 24 participants to their first CFTR modulator was evaluated at least 6 weeks after starting treatment. The mean increase in FEV1pp after treatment with LUM/IVA or TEZ/IVA was 3.56 percentage points (95% CI 0.40 to 6.72, online supplemental figure S2A). The mean decrease in SC after treatment was 21.8 mmol/L (95% CI 15.81 to 27.76, online supplemental figure S2B). Consistent with previous studies,^5 6^ there was marked heterogeneity in both FEV1pp and SC response to modulators between individual participants (figure 2).

This heterogeneous response was also demonstrated with subsequent modulator treatments (online supplemental file 2).

### Lack of identifiable relationship between additional *CFTR* variants and clinical response (FEV1pp and SC)

Given that additional variants alongside F508del could affect modulator effectiveness, we investigated these variants in our cohort.^37^ Sequencing of the *CFTR* gene confirmed that all 24 participants were homozygous for F508del and, in addition, identified 231 variants across 218 variable sites, from the *CFTR* promoter to the 3’untranslated region (online supplemental figure S3 and table S3). Phasing of these variants revealed 44 distinct haplotypes, which reduced to three haplotypes when considering coding sequence only. Twenty participants were homozygous for one haplotype, designated haplotype AA, three carried haplotype AB and one carried haplotype AC (online supplemental table S2). The *CFTR* variant L467F, previously reported to make F508del patients non-responsive to modulators, was not found in our cohort.^15 16 37^ No discernible patterns of clinical response as measured by FEV1pp and SC were observed with the haplotype (online supplemental table S2).

### Lack of identifiable relationship between pharmacogenomics and clinical response as measured by FEV1pp and SC

**Figure 3:**
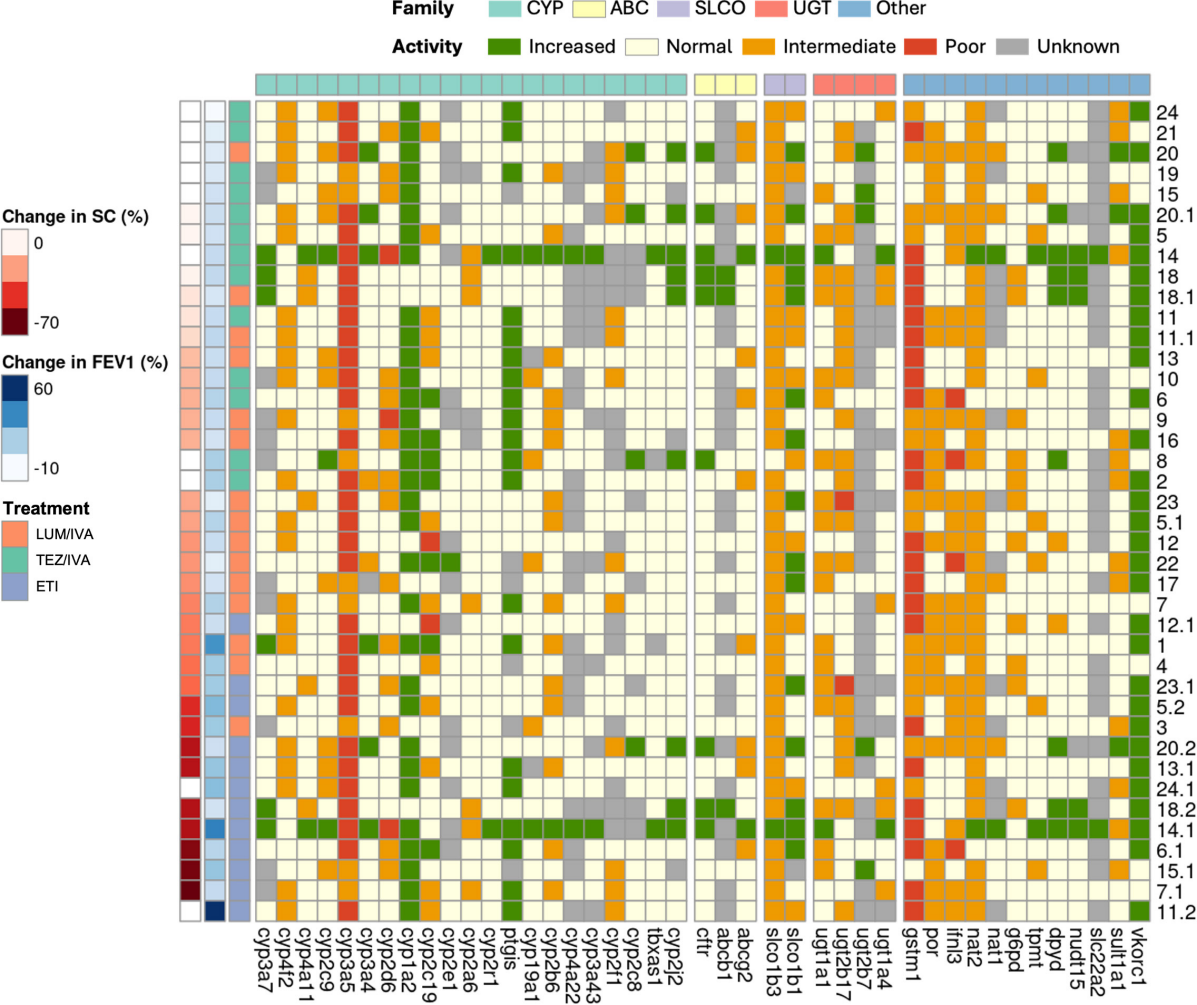
Pharmacogene profile across participants. The predicted activity levels of pharmacogenes were categorised for 58 genes from the cytochrome P450 enzymes (CYP), ATP-binding cassette transporters (ABC), solute carrier families (SLC and SLCO) and UDP-glucuronosyltransferases (UGT). Activity levels are indicated based on their Stargazer score; increased (> 2), normal (= 2), intermediate (1 - 2), poor (0–1) and unknown (< 0). Each column contains the predicted activity of a pharmacogene. Participants were ranked primarily by their responsiveness to treatment as measured by the relative increase in SC. FEV1pp was used to further rank participants with missing SC data. Each participant is represented once for each CFTR modulator they received. Pharmacogenes are not shown when the output for all participants was ‘normal’ or ‘unknown’. FEV1pp, forced expiratory volume in 1 s, percent predicted; SC, sweat chloride.

Since variability in drug metabolism and transporter activity can influence drug exposure,^13 14 38^ we performed a pharmacogenetic analysis of 58 genes. To overcome the limitation of a small available sample size and distinct enzyme activity labels, we used descriptive heatmaps to present the relationships between specific pharmacogenetic activity and treatment efficacy (figure 3). We identified considerable variability in pharmacogenes. No normal function phenotypes were observed for CYP3A5, whereas other genes displayed more variability, with CYP2C19 exhibiting phenotypes from every category.

A significant degree of variability was observed in the relationships between gene function and treatment efficacy. The number of altered genes was generally consistent across individuals, except in participant #14 who possessed a high number of genes with increased function (26). Trends only emerged with individual CFTR modulators. UGT2B17 exhibited a trend towards reduced function in non-responders to LUM/IVA and ETI but not TEZ/IVA (online supplemental figure S4A–C). GSTM1 function was more likely to be poor in responders to TEZ/IVA and ETI but not LUM/IVA (online supplemental figure S4A–C). CYP2C19 and CYP2C9 activity was inversely correlated with increased clinical response to TEZ/IVA (online supplemental figure S4B). This was not seen for LUM/IVA and ETI (online supplemental figure S4A,C). A trend towards increased activity of PTGIS in participants with the greatest response to ETI was demonstrated (online supplemental figure S4C).

### Heterogeneous in vitro functional response to CFTR modulators as measured by ΔIsc in differentiated-HNEC cultures

Differentiated-HNEC cultures created for each participant displayed pseudostratified epithelium with functional maturity, evident by mean ciliary beat frequency of 7.49 Hz and transepithelial electrical resistance of 432 Ω.cm^2^ (online supplemental figure S5).^39^ Baseline CFTR activity (ΔIsc) measured in participants differentiated-HNEC cultures was less than 10% of wild-type (WT) reference values (Mean 1.56%, 95% CI −0.21 to 3.33%) (online supplemental figure S6).

**Figure 4:**
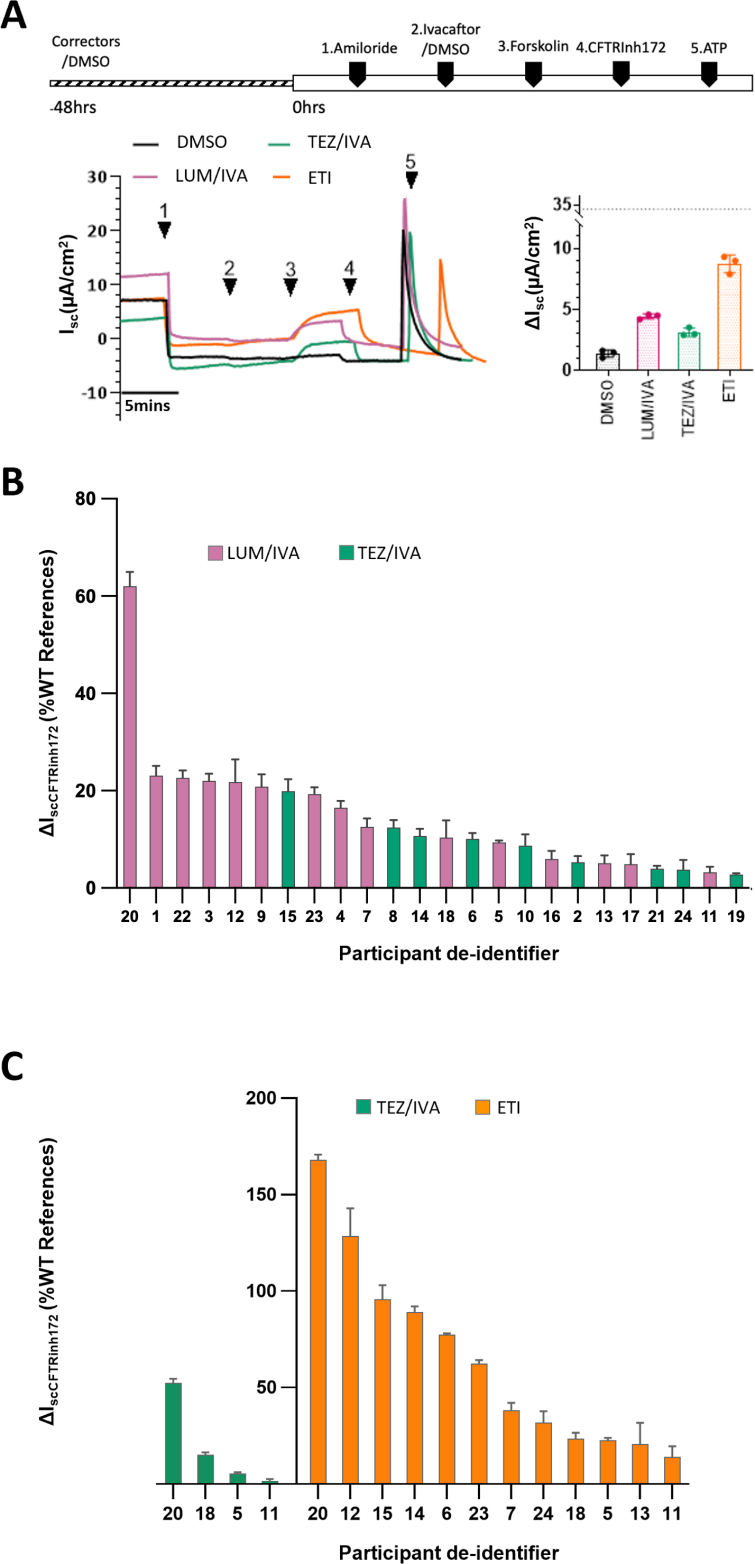
Heterogeneous in vitro functional response to CFTR modulators. (**A**) Representative recordings and results from an individual participant. Changes in short-circuit current (ΔIsc) in differentiated human nasal epithelial cell (HNEC) culture from study participant number five. Cells were either untreated (0.01% dimethyl sulfoxide (DMSO) vehicle) or pre-treated with a corrector(s) (lumacaftor (LUM), tezacaftor (TEZ:T) or elexacaftor (**E**) plus TEZ for 48 hours). Functional CFTR expression was then measured by sequentially adding 100 µM apical amiloride ((1) amiloride), 0.01% apical DMSO vehicle control or 10 µM apical VX-770 (ivacaftor (IVA;I)), followed by 10 µM basal forskolin ((3) forskolin), 30 µM apical CFTR inhibitor ((4) CFTRinh172) and 100 µM apical ATP ((5) ATP). The basolateral-to-apical chloride gradient was used to measure functional CFTR activity. The in vitro ΔIsc results for participant number five are displayed on the bar chart, with the dotted line indicating the wild-type reference level for our lab (as reported in Wong *et al*^39^). CFTR activity is expressed as the inhibition of Isc by CFTRinh172 after activation by forskolin. Each dot represents an independent HNEC culture. Data are presented as mean±SEM. (**B**) ΔIsc in differentiated HNEC cultures to first modulator treatment. Bar chart showing ΔIsc (% of wild-type reference) in participants differentiated HNEC cultures in response to their first CFTR modulator treatment. CFTR activity is expressed as the inhibition of Isc by CFTRinh172 after activation by forskolin. Modulator response is calculated by subtracting baseline CFTR activity (DMSO) from modulator-treated results and reported as a percentage of normal (wild type). (**C**) ΔIsc in differentiated HNEC cultures to subsequent modulator treatments. Bar chart showing ΔIsc (% of wild-type reference) in response to a subsequent CFTR modulator regimen. CFTR activity is expressed as the inhibition of Isc by CFTRinh172 after activation by forskolin. Changes are calculated by subtracting baseline CFTR activity (DMSO) from modulator-treated results and reported as a percentage of normal (wild type). Each bar represents the mean result of three replicate cultures per participant. Error bars represent the SEM.

When participants’ differentiated-HNEC cultures were treated with the CFTR modulators corresponding to those received as part of their routine CF care (online supplemental table S2), the mean increase in ΔIsc was 17.31% of WT (95% CI 9.36 to 25.26) for LUM/IVA, 11.69% of WT (95% CI 3.66 to 19.73) for TEZ/IVA and 64.28% of WT (95%CI 33.26 to 95.30) for ETI. Individual ΔIsc results for each participant highlight the heterogeneity in response to CFTR modulators between participants, independent of which modulator was used (figure 4B,C).

### Linear relationship between in vitro functional response and in vivo clinical response to the first CFTR modulator

**Figure 5:**
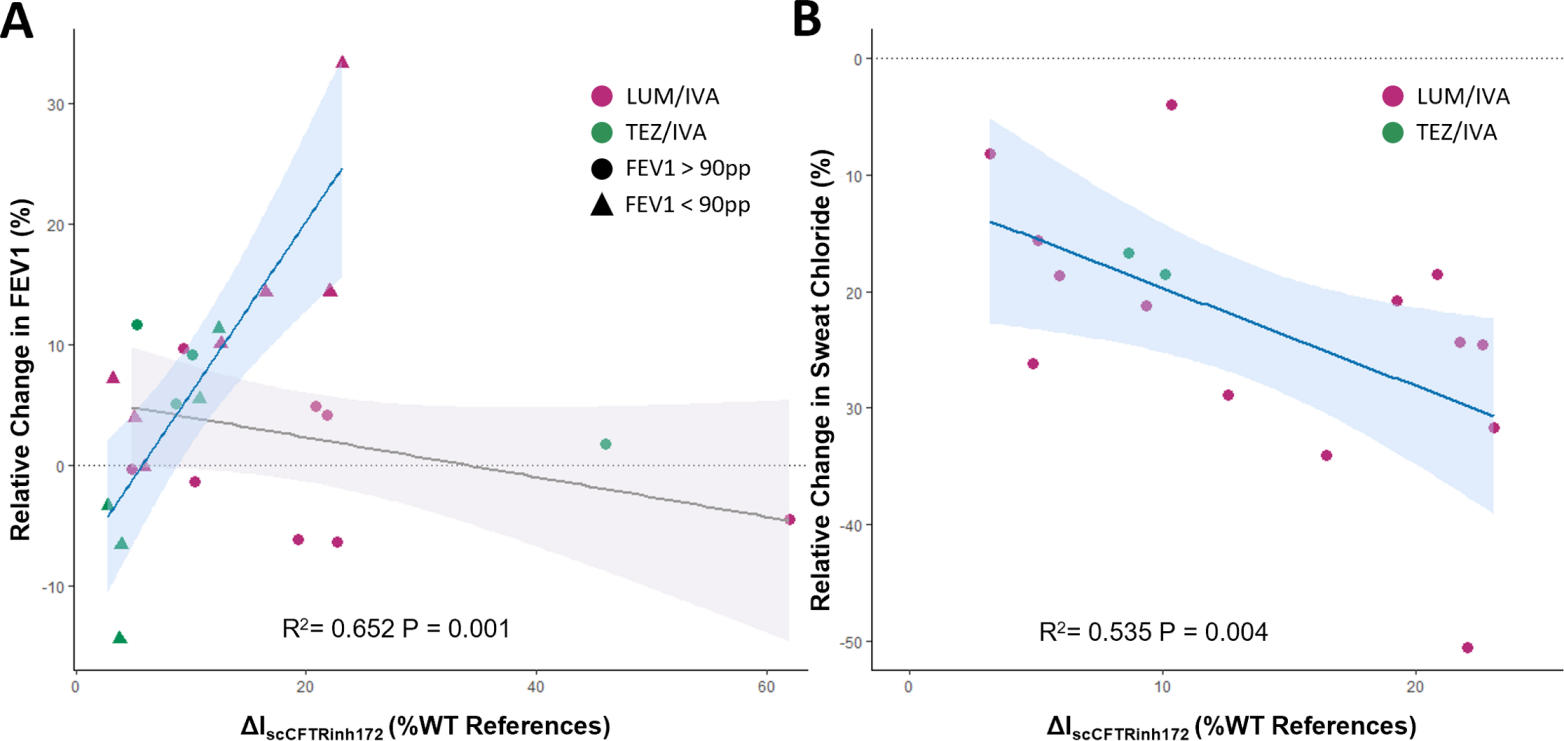
Comparison of in vitro and in vivo CFTR modulator responses to the first modulator taken by participants. (**A**) FEV1pp scatterplot. Relative change in FEV1pp is plotted against the change in short circuit current (ΔIsc) in differentiated-HNEC cultures in response to CFTR Inh172, as percentage of the wild type reference response. Each point represents an individual participant, denoted by dots (n=12) indicating baseline FEV1pp above 90 or triangles (n=12) indicating baseline FEV1 less than 90. Data are analysed using multiple linear regression. Separate regression lines are shown for participants with baseline FEV1 above and below 90 pp. The colour of each point represents the specific CFTR modulator treatment administered. (**B**) SC scatterplot. Relative change in SC is plotted against ΔIsc in differentiated-HNEC cultures in response to CFTR Inh172, as a percentage of the wild type reference response. Each dot represents a participant (n=16). Data are analysed using multiple linear regression. The colour of each point represents the specific CFTR modulator treatment administered. The shaded area indicates the 95% CI of the regression line. FEV1pp, forced expiratory volume in 1 s, percent predicted; HNEC, human nasal epithelial cell; IVA, ivacaftor; LUM, lumacaftor; SC, sweat chloride; TEZ, tezacaftor.

Multiple linear regression was conducted to examine the relationship between the in vitro functional response (ΔIsc) in differentiated HNEC cultures and the participant’s in vivo clinical response (FEV1pp, SC) to their first CFTR modulator. A significant linear relationship was found between participants’ ΔIsc and their change in FEV1pp, with strong evidence of an interaction with baseline FEV1pp group (interaction p value <0.001, model R^2^=0.652; figure 5A, online supplemental table S4). Additionally, a significant linear relationship was identified between ΔIsc and the SC response to CFTR modulator treatment (b=1.110, 95% CI 0.423 to 1.798, p=0.004, Model R^2^=0.535, figure 5B).

### Predictive capacity of in vitro functional response to the in vivo clinical response to the first CFTR modulator

**Figure 6:**
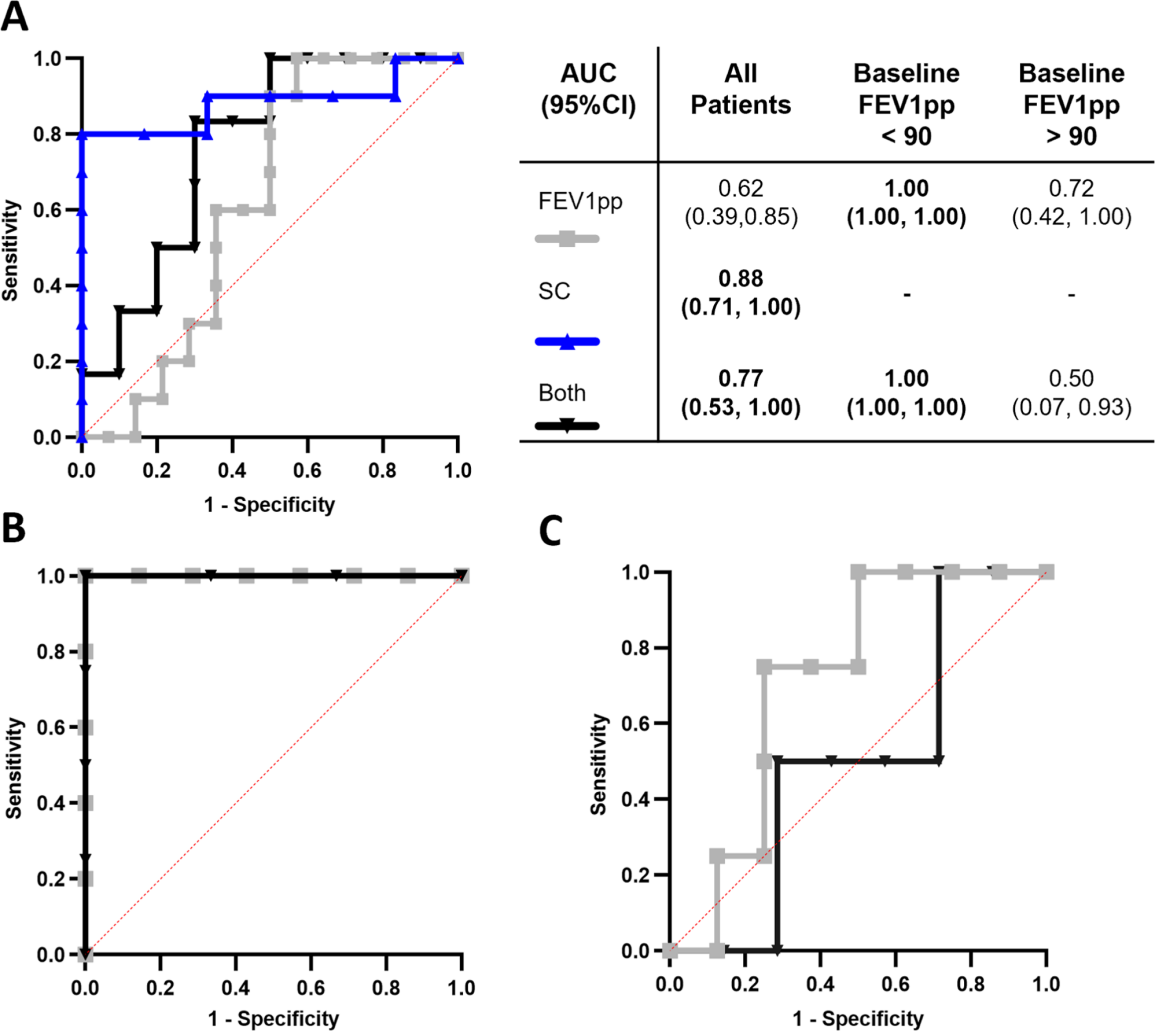
Receiver operator characteristic (ROC) curves of in vitro drug effect predicting in vivo clinical response. (**A**) ROC curves for all participants. The predictive value of in vitro measurements for overall clinical responses. The grey curve represents FEV1pp (n=24), while the blue curve represents SC (n=16). The black curve shows the predictive value of a significant change in both FEV1pp and SC (n=16). The diagonal red line represents the line of no discrimination. The table on the right provides the area under the curve (AUC) values with their 95% CIs for different participant groups. Statistically significant results are highlighted in bold. (**B**) ROC curves for participants with baseline FEV1pp under 90. The grey curve represents FEV1pp (n=12), and the black curve shows the predictive value of a change in both FEV1pp and SC (n=7). (C) ROC curves for participants with baseline FEV1pp above 90. The grey curve represents FEV1pp (n=12), and the black curve shows the predictive value of a change in both FEV1pp and SC (n=9). Clinical response criteria: increase in FEV1pp was considered positive when FEV1pp increased by at least 5 percentage points. A decrease in SC was considered positive when SC was reduced by at least 20 mmol/L. FEV1pp, forced expiratory volume in 1 s, percent predicted; SC, sweat chloride.

To assess the capacity of differentiated-HNEC cultures to identify a predefined clinically significant response (FEV1pp increase >5 percentage points, SC decrease >20 mmol/L) to treatment, we used ROC curves (figure 6). When examining the data as a whole, curves incorporating a decrease in SC demonstrated high predictive accuracy (area under the curve (AUC) 0.88 (95% CI 0.71 to 1.00) and AUC 0.77 (95% CI 0.53 to 1.00), figure 6A). Notably, when stratifying the data based on baseline FEV1pp being above or below 90, significant predicative accuracy was only observed in the group with a baseline FEV1pp below 90 (AUC 1.00 (95% CI 1.00 to 1.00)) for both FEV1pp response and combined FEV1pp and SC response, although we note the group size was small (figure 6B,C).

### Confirmation of trends with exploratory analysis of subsequent modulator treatments

**Figure 7:**
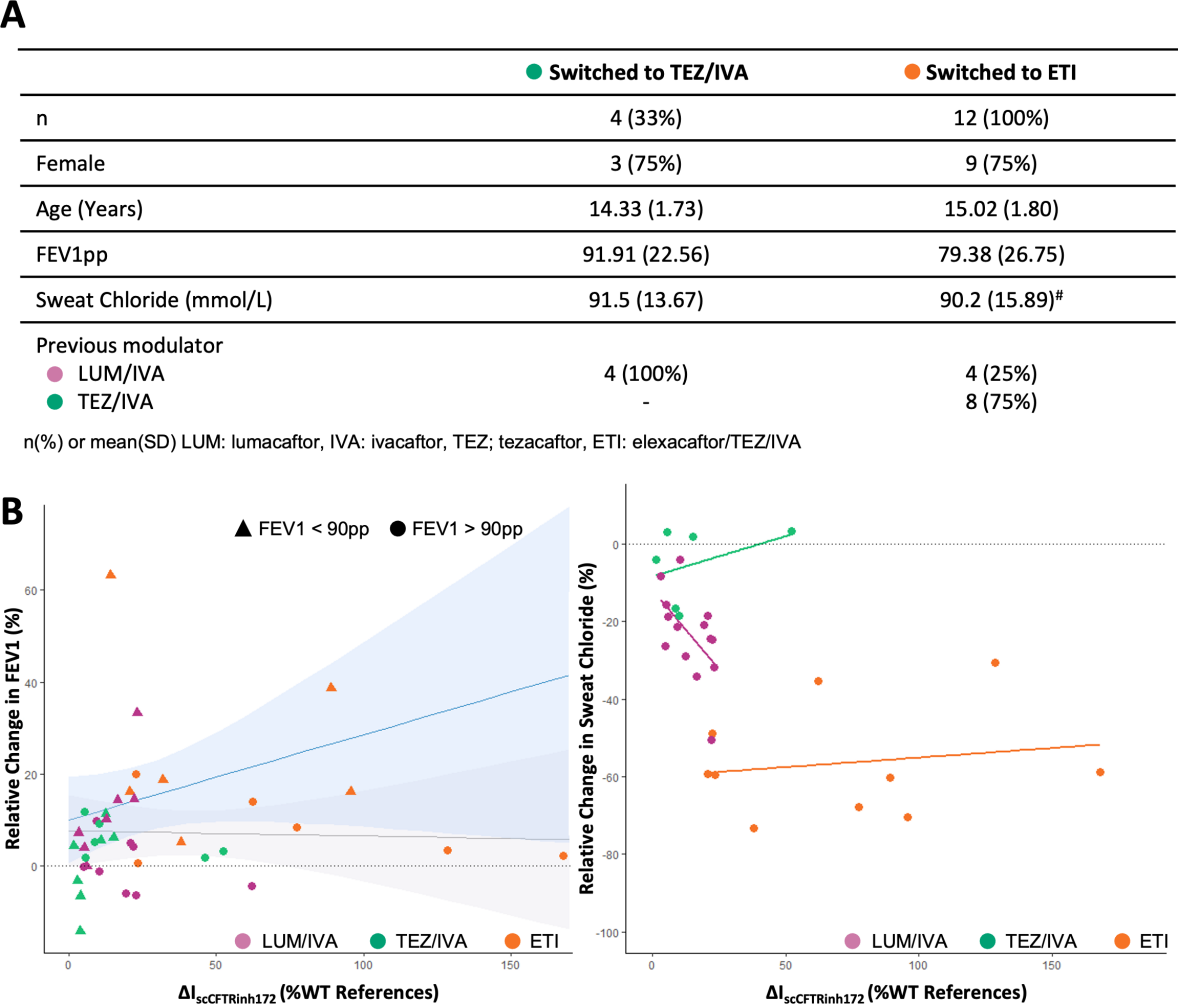
Exploratory analysis of the relationship between CFTR drug response in vitro and in vivo, including subsequent modulator treatments. (**A**) Patient characteristics. The table summarises the characteristics of participants who switched to either tezacaftor/ivacaftor (TEZ/IVA) or elexacaftor/TEZ/IVA (ETI) treatments. Values for FEV1pp and SC are recorded immediately before switching treatments. Symbol (#) denotes three participants did not have measurements before switching to ETI. (**B**) FEV1pp versus CFTR activity (ΔIsc) in differentiated HNEC cultures. Plots of relative change in FEV1pp vs ΔIsc in response to CFTRinh172 treatment. Separate regression lines are shown for participants with baseline FEV1 above and below 90pp. The shaded area indicates the 95% CI. Each point (dot/triangle) represents a single comparison (n=40). The colour of each point represents the specific CFTR modulator treatment administered. (C) Scatterplot of SC vs change in in vitro CFTR activity (ΔIsc) in differentiated-HNEC cultures. Relative change in SC is plotted against the ΔIsc in response to CFTR inh172, as a percentage of the wildtype reference response. Each dot represents a single comparison (n=30 due to missing SC data for nine participants (online supplemental table S2)). Data are analysed using generalised estimating equations with a variance components covariance structure to account for repeated measures. The colour of each point and regression line represents the specific CFTR modulator treatment administered. FEV1pp, forced expiratory volume in 1 s, percent predicted; HNEC, human nasal epithelial cell; SC, sweat chloride.

To further investigate the consistency of the differentiated-HNEC culture responses with in vivo clinical outcomes, we conducted exploratory analysis on participants who underwent changes in their CFTR modulator treatments. This analysis included 40 paired in vivo–in vitro results (table 1, figure 7A). Using generalised estimating equations (GEE), we found strong evidence that the ΔIsc predicted the change in FEV1pp (p=0.006, online supplemental table S5A). Consistent with the primary analysis, baseline FEV1pp group significantly influenced the change in FEV1pp (p=0.028, figure 7B, online supplemental table S5B). We additionally observed that the choice of CFTR modulator significantly affects the change in FEV1pp (p=0.044, online supplemental table S5B), and the relationship between ΔIsc and change in FEV1pp varied by CFTR modulator (p=0.030, online supplemental figure S7A). Sensitivity analysis, after removing two outliers identified by checking normality of residuals after linear fit (P.d #1 and #11), showed strong evidence that ΔIsc predicts change in FEV1pp (p<0.001) and confirmed an interaction with baseline FEV1pp group (p=0.001) (online supplemental figure S7B and table S5B).

Finally, we explored the relationship between ΔIsc and change in SC (with 30 out of 40 comparisons available). Although the initial analysis did not find a significant relationship between the ΔIsc and SC measurements (p=0.441, online supplemental figure S7C), further analysis indicated that the trend varied significantly by CFTR modulator (p <0.0001, online supplemental figure S7C). After excluding the CFTR modulator variable from the statistical model and including age and gender variables only, we found strong evidence that the ΔIsc predicted change in SC (p=0.008, online supplemental table S5C and figure S7D).

## Discussion

Our findings support the role of personalised medicine in CF, recognising that treatment response is influenced by complex biological variability, including epithelial function, baseline lung function and age.^1 17^ We demonstrated the predictive value of differentiated-HNEC cultures, particularly in identifying significant reduction in SC levels (> 20 mmol/L) and improvement in FEV1pp (> 5 percentage points) in participants with baseline FEV1pp below 90. While no predefined cut-off was used to dichotomise in vitro responses, exploratory analysis using a conservative threshold of <10% WT CFTR function (ΔIsc), as described in prior studies,^24 31^ would have identified five individuals as in vitro non-responders to their first modulator. Of these, one participant (#5, DIsc=9.4% WT) experienced a 10 percentage point increase in FEV1pp, highlighting the limitations of rigid thresholds and biological variability near the margin. Inclusion of additional clinical endpoints, such as exacerbation rate, body mass index and quality of life scores, could aid in better identification of non-responders.

The variable response to CFTR modulators, highlighted by our secondary analysis involving subsequent treatments, emphasises the complexities in drug efficacy. Extending our analysis to include subsequent modulator treatments for 12 participants, we found that the predictive capability of differentiated-HNEC cultures remained robust, though variation across different CFTR modulators was observed in SC response. The high inter-individual variability in CFTR rescue with ETI (40%–150% WT, online supplemental figure S7) highlights the influence of cellular and molecular context on modulator efficacy, underscoring the potential value of functional testing for individualised treatment decisions.

In addition, we sought to ascertain if the heterogeneous response to CFTR modulator treatments could be explained by additional variants. Specific allele combinations in cis have been shown to negatively impact the efficacy of CFTR modulators.^15 16 40^ However, despite identifying 231 variants beyond F508del, no association with treatment response was found. We identified individual participants with variable response to different modulators (online supplemental figure S2); however, no specific haplotype was associated with variable response. Three participants carried the complex allele I1027T;F508del (haplotype AB).^15 41^ Although this allele did not compromise the response to ETI in our participants, only one of the three showed clinical improvement with TEZ/IVA treatment. Reports of PwCF carrying the I1027T;F508del are sparse but suggest no impairment in CFTR modulator response.^10 15 37^ Our data suggest that *CFTR* genotype alone does not fully predict drug response due to the influence of various factors, including genetic modifiers and individual variations in drug metabolism.^38 42^

We extended our genomic analysis to predict the activity of participants’ drug metabolising enzymes (pharmacogenes). Although our analysis of these pharmacogenes was limited by the small sample size available, some trends were observed. Differences in the predicted activity of CYP450 enzymes involved in CFTR modulator metabolism did not correlate with changes in FEV1pp and SC. Furthermore, trends differed between CFTR modulators. A potential inverse correlation between CYP2C9 and CYP2C19 activity scores and clinical response to TEZ/IVA was observed, but further statistical analysis was precluded by the small sample size. Additionally, this trend was not substantiated by available drug level data, which were limited by inconsistent timing, fasting status and missing covariate information. These exploratory findings require confirmation in larger, prospectively designed studies that can control for these variables. As such, the inconsistency in relationships between pharmacogene status and treatment efficacy complicates the identification of reliable genetic biomarkers for predicting positive drug responses across CFTR therapies. Future studies should strive to investigate pharmacogenes in larger participant cohorts.

Our study contributes valuable insights into the utility of differentiated-HNEC cultures in paediatric CF treatment, particularly for those homozygous for the F508del*-CFTR* variant. These models demonstrate their applicability across various CFTR modulators,^24 28 30^ highlighting their potential in aiding the selection of appropriate modulator therapy in a clinical setting. Differentiated-HNEC cultures could be used to identify non-responders to prevent exposure to side effects and unnecessary cost to patients and/or health systems by the use of an ineffective treatment.^7^ However, the observed plateau effect in clinical responses (FEV1pp or SC), despite maximal correction of CFTR function as achieved by ETI, necessitates additional consideration of the CF treatment paradigm. This effect, suggestive of a limit to the clinical benefits achievable with modulator therapy, was noted in our analysis of ETI data, resonating with recent findings that propose a ceiling treatment efficacy at approximately 30% CFTR protein function correction.^28^ This phenomenon indicates the need for alternative clinical measures or endpoints in clinical trials and care strategies, particularly for potent modulators like ETI, where traditional non-sensitive endpoints, such as spirometry, may not capture the full spectrum of patient benefits. In particular, lung clearance index (LCI), chest CT and MRI are emerging as more sensitive endpoints of lung disease in PwCF with preserved lung function and should be considered in future studies of CFTR modulator efficacy in paediatric cohorts.

Addressing the limitations of our study, including the pragmatic challenges of data collection in a paediatric clinical setting, we acknowledge the need for comprehensive and systematic approaches to data gathering. This is particularly critical in the context of SC measurements, given the intra-individual variability of SC levels (up to 18 mmol/L) and the sensitivity to short-term adherence to CFTR modulator.^8^ Future research should strive for completeness and accuracy in clinical data to better understand the nuances of modulator efficacy and patient response. Similarly, the in vitro studies may benefit from further standardisation; for example, various concentrations of CFTR modulators are tested when used alone or in combination,^3 16 33^ which may impact the magnitude of CFTR functional response. A global consensus on the drug concentration used in experiments will be advantageous to compare data across laboratories. Our exploratory analysis, including all modulator treatments, is limited by the low number of participants treated with ETI, due to clinical management decisions or transition to adult care, which precluded a definitive analysis of the correlation between in vitro CFTR function and clinical response for this regimen. Future studies are needed to validate functional testing specifically for ETI selection.

## Conclusion

As more CFTR modulators become available for patients to choose from,^7^ identifying the right drug for each patient will become increasingly challenging for CF clinicians. Our study highlights the heterogeneity of response to CFTR modulators in children and adolescents and underscores that response to one agent may not predict response to others in the same class. Studies of PwCF with FEV1pp above 90 or those unable to perform spirometry will require alternative clinical endpoints to evaluate their lung disease response to modulator treatment. Screening our cohort did not identify any *CFTR*-genotypic reason for non-response to treatment. Thus, a combination of genetic and functional testing may provide a more optimal approach for managing complex diseases such as CF.

## Supplementary material

10.1136/thorax-2025-223153 online supplemental file 1

10.1136/thorax-2025-223153 online supplemental file 2

10.1136/thorax-2025-223153 online supplemental file 3

## Data Availability

All data relevant to the study are included in the article or uploaded as supplementary information.
