# Supplementary material for: Predictive capacity of paediatric nasal epithelial cells in sequential CFTR modulator therapy
Source: Thorax. 2025 Dec 7;81(6):e223153. doi: 10.1136/thorax-2025-223153 (PMC13217057; doi:10.1136/thorax-2025-223153)
Supplement: online supplemental file 1 [file thorax-81-6-s001.docx]

Supplementary File 1: Figures and Tables.

**Table S1: Summary of studies undertaking comparative analysis of in *vivo in vitro* responses to CFTR modulators using differentiated-HNEC cultures**

| **Publication** | **Participant**  **age group** | **Participant**  **genotypes** | **Participant**  **modulator therapy** | **Results** |
| --- | --- | --- | --- | --- |
| Pranke I et al, 2017 [1] | > 12 years | F508del homozygous  (n = 7)  F508del/ S549N  (n = 1) | LUM/IVA  IVA | **FEV1pp**  R^2^ = 0.95 p < 0.0001 |
| McGarry M et al, 2017 [2] | >16 years | Various;  (n = 7)  F508del/1154insTC; G542X/3849þ10kbC->T; F508del/Y563N; R334W/681delC; 1717-1G->A/G85E; 1717-1G->A/A455E; F508del/A455E | IVA | **SC**  Individual results reported |
| Pranke I et al, 2019 [3] | > 12 years | F508del homozygous  (n = 16) | LUM/IVA | **FEV1pp**  r = 0.735 p = 0.004 |
| Debley JS et al, 2020 [4] | > 18 Years | R117H; G551D  (n = 7) | IVA | **FEV1pp**  r = 0.71 R^2^ = 0.51 P = 0.05  **SC**  r = 0.85 R^2^ = 0.72 P < 0.02 |
| Dreano E et al, 2023[5] | 11 Children (median 12 years)  28 adults (> 18 years) | Various; (n = 39)  I601F/621+2T>G; R347P/N1303K; G85E/G85E (n = 2); S492F/R1066C; G85E/2622+1G>A; R74W;V201M;D1270N/711+1G>T; M1101K/3396delC; S977F;TG12T5/S977F;TG12T5; H1085R/N1303K (n=2); I507del/711+1G>T; N1303K/G542X; N1303K/N1303K; N1303K/CFTRdele3-10,14b-16; N1303K/R1162X; 1717-1G>A/3659delC; 1717-1G>A/2183AA>G; R1066C/E585X; Q552P/711+1G>T; G542X/1717-1G>A; 357delC/357delC; CFTRdele19/CFTRdele19; G542X/4271delC; 2183AA>G/2183AA>G; M1T/M1T; W1282X/1078delT; 4096-3C>G/4096-3C>G; R334W/M1T; 4374+1G>A/4374+1G>A; Q493X/2183AA>G; 711+1G>T/E1104X; 1525-1G>A/1525-1G>A; 711+1G>T/711+1G>T; R553X/1717-1G>A; L558S/2183AA>G; W1063X/W1063X; 2789+5G>A/K710X; 1525-1G>A/1525-1G>A | ETI | **FEV1pp**  ρ = 0.787, p < 0.0001.  A non-linear relationship was observed overall.  In samples with ⩽12%, correction, the relationship between CFTR correction level and FEV1 % pred improvement was linear  (ρ = 0.707, p < 0.0001)  **SC**  ρ = −0.687, p < 0.0001  A non-linear relationship was observed overall.  In samples with <10%, correction, the relationship between CFTR correction level and SC improvement was linear  (ρ = −0.486, p = 0.009). |
| Ratjen F et al, 2025 [6] | Mean age 23.1± 12.1 | G551D (n=2),  F508del homozygous (n=36),  F508del heterozygous (n=7); G542X; N1303K; I507; D1152H; G628R; 457TATdelinsG; T1246 | IVA  LUM/IVA  TEZ/IVA  ETI | **FEV1pp**  r = 0.462 (0.232-0.644)  **LCI**  r = -0.483 (-0.733- -0.118)  **SC**  r = -0.375 (-0.592- -0.108)  **CFQ-R**  r = 0.275 (-0.036-0.537) |

FEV1pp: Forced Expiratory Volume in 1 second, Percent Predicted. SC: Sweat Chloride

1. Pranke IM, Hatton A, Simonin J, et al. Correction of CFTR function in nasal epithelial cells from cystic fibrosis patients predicts improvement of respiratory function by CFTR modulators. Scientific Reports; 2017 2. McGarry ME, Illek B, Ly NP, et al. In vivo and in vitro ivacaftor response in cystic fibrosis patients with residual CFTR function: N-of-1 studies. Pediatr Pulmonol; 2017

3. Pranke I, Hatton A, Masson A, et al. Might brushed nasal cells be a surrogate for CFTR modulator clinical response? Am J Respir Crit Care Med American Thoracic Society; 2019

4. Debley JS, Barrow KA, Rich LM, et al. Correlation between ivacaftor-induced CFTR activation in airway epithelial cells and improved lung function: A proof-of-concept study. Ann Am Thorac Soc American Thoracic Society; 2020

5. Dreano E, Burgel PR, Hatton A, et al. Theratyping cystic fibrosis patients to guide elexacaftor/tezacaftor/ivacaftor out-of-label prescription. Eur Respir J; 2023

6. Ratjen F, Stanojevic S, Gunawardena T , et al. Relationship between theratyping in nasal epithelial cells and clinical outcomes in people with cystic fibrosis. Eur Respir J; 2025

**Table S2: Individual study participants demographics, clinical and *in vitro* response to CFTR modulators.**

| **Participant de-identifier number** | **Age at baseline**  **(years)** | **Baseline FEV1pp*** | **Baseline SC (mmol/L)** | **Drug treatments** | **Post treatment FEV1pp*** | **Post treatment SC (mmol/L)** | **Fsk response (%WT)** | **CFTR inh response (%WT)** | **Haplotype**** |
| --- | --- | --- | --- | --- | --- | --- | --- | --- | --- |
| 1 | 7.3 | 74 | 104 | LUM/IVA | 99 | 71 | 36.1 | 23.12 | AA |
| 2 | 13.5 | 104 | NA | TEZ/IVA | 117 | NA | 11.76 | 5.33 | AB |
| 3 | 17.4 | 83 | 99 | LUM/IVA | 95 | 49 | 22.49 | 22.06 | AA |
| 4 | 15.7 | 71 | 97 | LUM/IVA | 81 | 64 | 8.46 | 16.48 | AA |
| 5 | 14.3  (15.3)  (17.0) | 99  (98)  (95) | 94 | LUM/IVA  TEZ/IVA  ETI | 109  100  114 | 74  97  48 | 14.63  17.36  27.81 | 9.37  5.41  22.57 | AA |
| 6 | 13.7  (14.9) | 103  (107) | 81 | TEZ/IVA  ETI | 112  116 | 66  26 | 19.31  127.9 | 10.11  77.44 | AA |
| 7 | 9.6  (12.6) | 74  (67) | 97 | LUM/IVA  ETI | 81  70 | 69  26 | 19.34  37.91 | 12.59  38.02 | AA |
| 8 | 16.1 | 64 | 103 | TEZ/IVA | 71 | NA | 10.99 | 12.4 | AA |
| 9 | 5.5 | 108 | 113 | LUM/IVA | 113 | 92 | 11.58 | 20.86 | AA |
| 10 | 13.5 | 93 | 66^#^ | TEZ/IVA | 98 | 55 | 1.61 | 8.67 | AA |
| 11 | 13.8  (16.0)  (17.5) | 60  (46)  (39) | 98 | LUM/IVA  TEZ/IVA  ETI | 64  48  63 | 90  94  NA | 4.42  5.24  15.35 | 3.2  1.56  14.12 | AB |
| 12 | 11.9  (15.2) | 100  (99) | 111 | LUM/IVA  ETI | 104  102 | 84  77 | 24.81  126.06 | 21.78  128.58 | AA |
| 13 | 11.3  (14.7) | 88  (87) | 96 | LUM/IVA  ETI | 92  101 | 81  39 | 2.24  15.41 | 5.08  20.68 | AA |
| 14 | 14.2  (16.0) | 47  (41) | 98^#^ | TEZ/IVA  ETI | 50  56 | NA  39 | 19.41  103.62 | 10.73  89.04 | AA |
| 15 | 12.1  (13.0) | 93  (86) | 110 | TEZ/IVA  ETI | 94  100 | NA  34 | 30.02  132.75 | 19.92  95.67 | AA |
| 16 | 15.8 | 68 | 102 | LUM/IVA | 68 | 83 | 12.86 | 5.94 | AC |
| 17 | 6.1 | 105 | 84 | LUM/IVA | 105 | 62 | 8.19 | 4.89 | AA |
| 18 | 10.0  (12.0)  (12.5) | 97  (89)  (91) | 99 | LUM/IVA  TEZ/IVA  ETI | 96  94  91 | 95  101  40 | 23.2  21.51  45.95 | 10.35  15.16  23.36 | AA |
| 19 | 15.8 | 78 | NA | TEZ/IVA | 75 | NA | 3.83 | 2.77 | AA |
| 20 | 13.7  (14.0)  (16.3) | 112  (112)  (106) | 92 | LUM/IVA  TEZ/IVA  ETI | 107  116  108 | NA  95  38 | 103.3  88.25  256.45 | 62  52.25  167.87 | AA |
| 21 | 15.2 | 77 | NA | TEZ/IVA | 72 | NA | 1.53 | 3.94 | AA |
| 22 | 8.3 | 100 | 106 | LUM/IVA | 93 | 80 | 22.35 | 22.64 | AA |
| 23 | 11.0  (13.4) | 105  (100) | 96 | LUM/IVA  ETI | 99  114 | 76  62 | 36.84  69.93 | 19.27  62.22 | AA |
| 24 | 17.0  (17.5) | 46  (37) | NA | TEZ/IVA  ETI | 39  44 | NA  NA | 8.96  38.75 | 3.77  31.81 | AB |

*FEV1pp is based on GLI reference. **Haplotype A: c.1210-13G>T/V470M/F508del. B: c.1210-13G>T /V470M/F508del/I1027T. C: c.1210-13G>T/V470M/F508del/c.*133del.

( )subsequent baseline prior to changing modulator.  ^#^Neonatal sweat chloride used. FEV1pp: Forced Expiratory Volume in 1 second, Percent Predicted. SC: Sweat Chloride NA: Not available


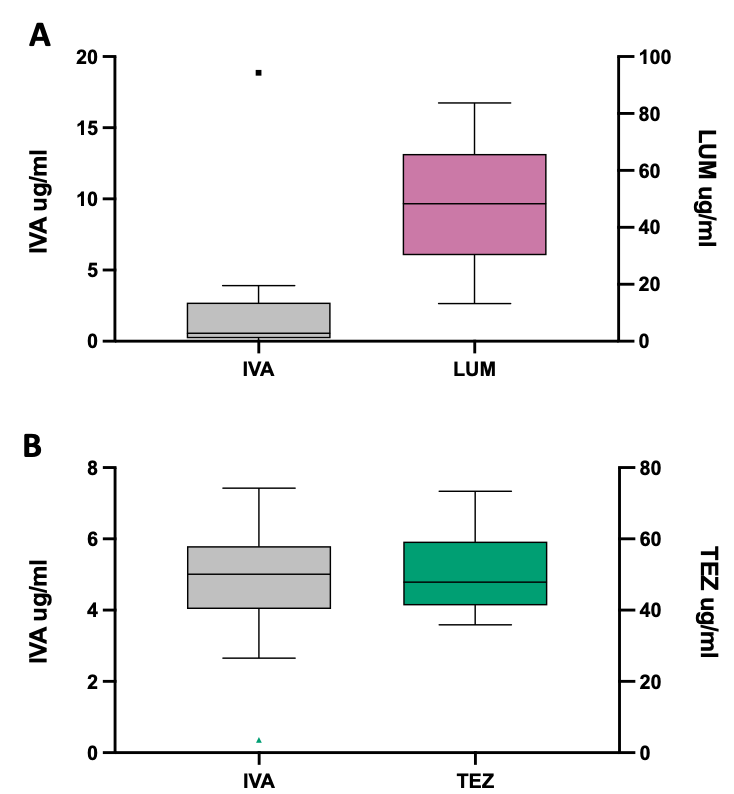


**Figure S1: CFTR modulator levels from participants measured by LC-MS**​. CFTR modulator levels measured in opportunistically obtained blood samples from 14 study participants. Three participants had repeat samples from multiple time points. **A)** Ivacaftor (IVA) and lumacaftor (LUM) levels for participants on LUM/IVA (n = 7). **B)** IVA and Tezacaftor (TEZ) levels (n=7). IVA levels are plotted on the left y axis and LUM or TEZ drug levels are plotted on the right y axis. The centre line of each box blot denotes the median value. The box denotes the 25th and 75th percentiles of the dataset. The whiskers are as per Tukey formula. Any values beyond these upper and lower bounds are considered outliers and shown as a single point.


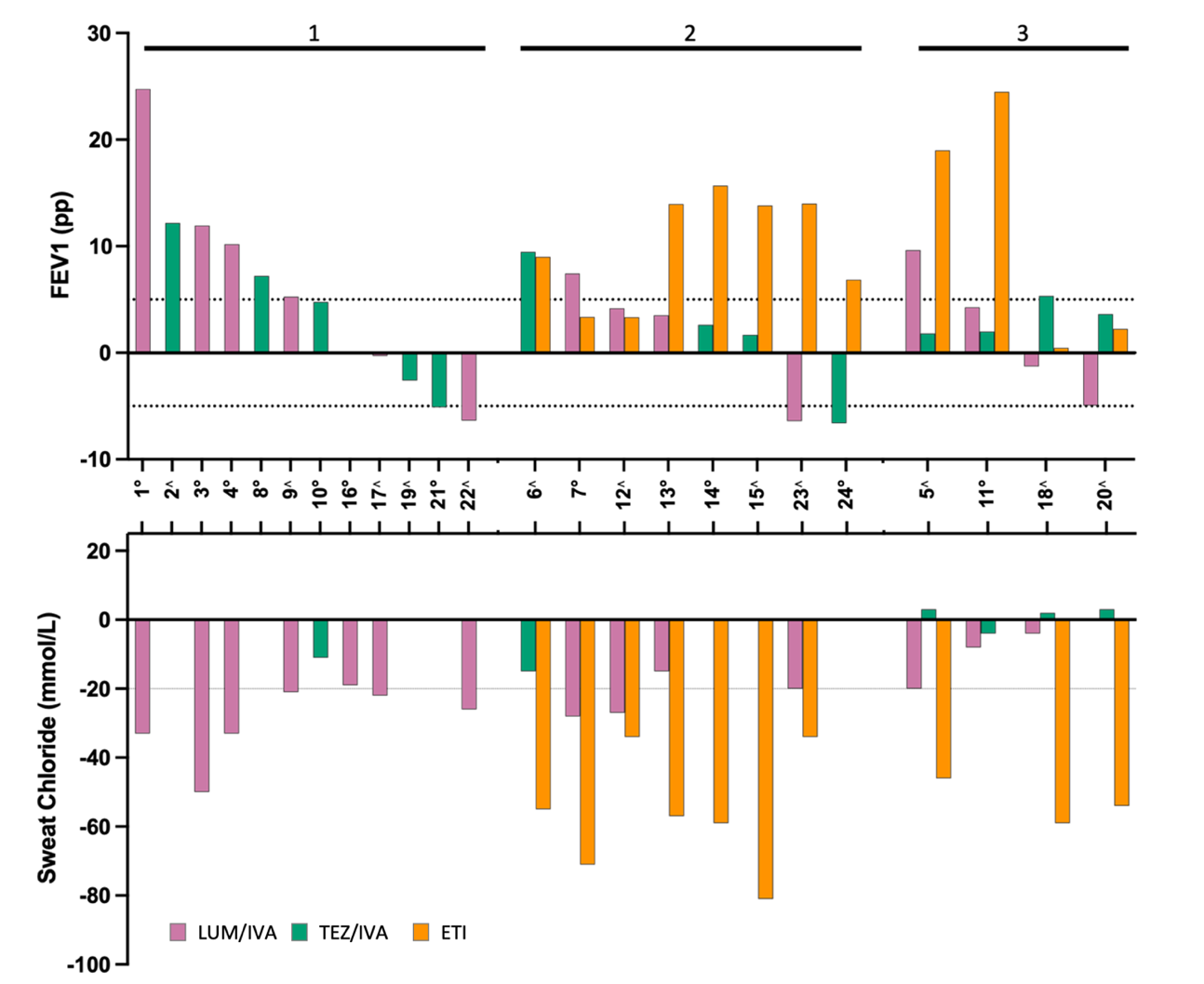

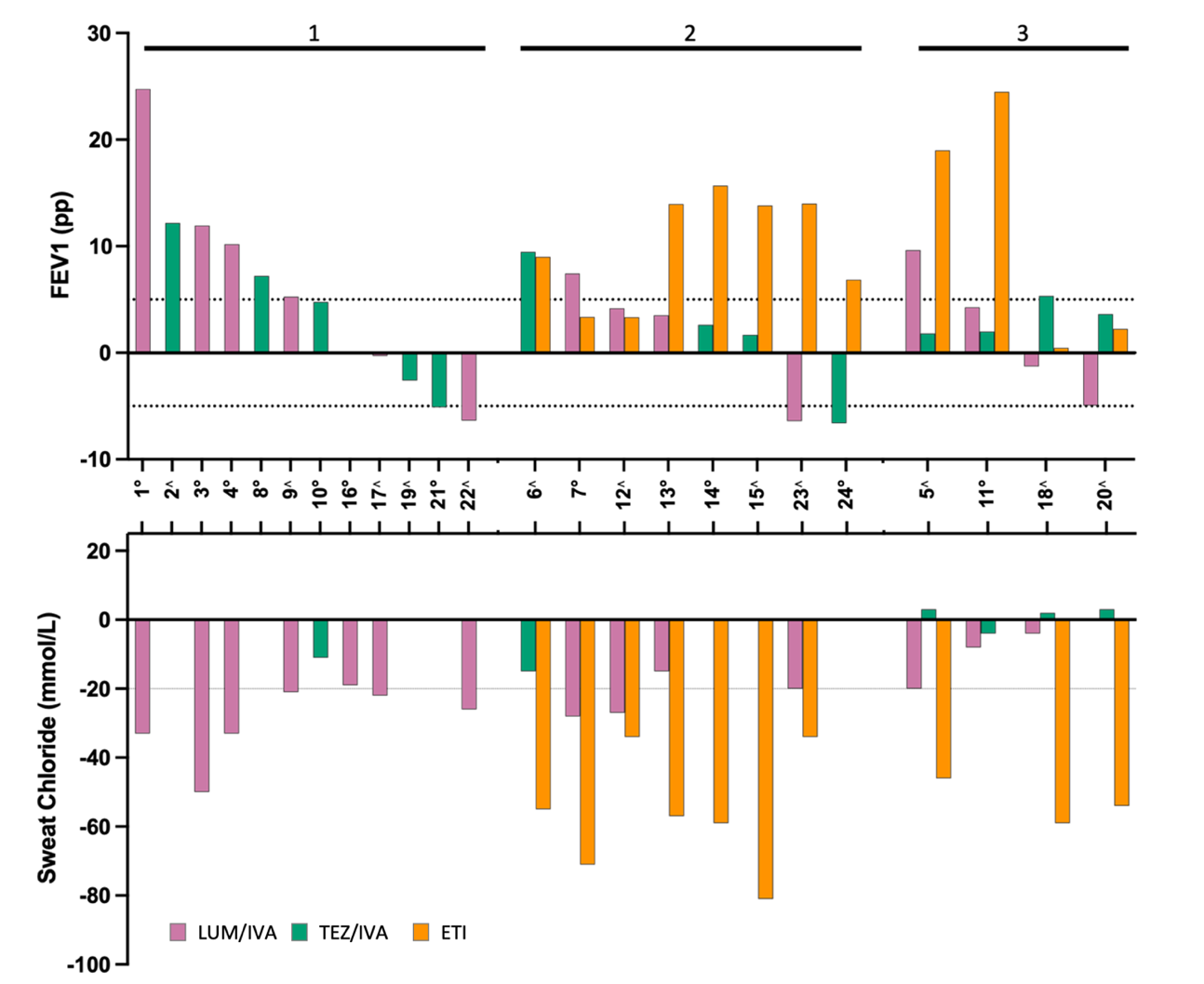

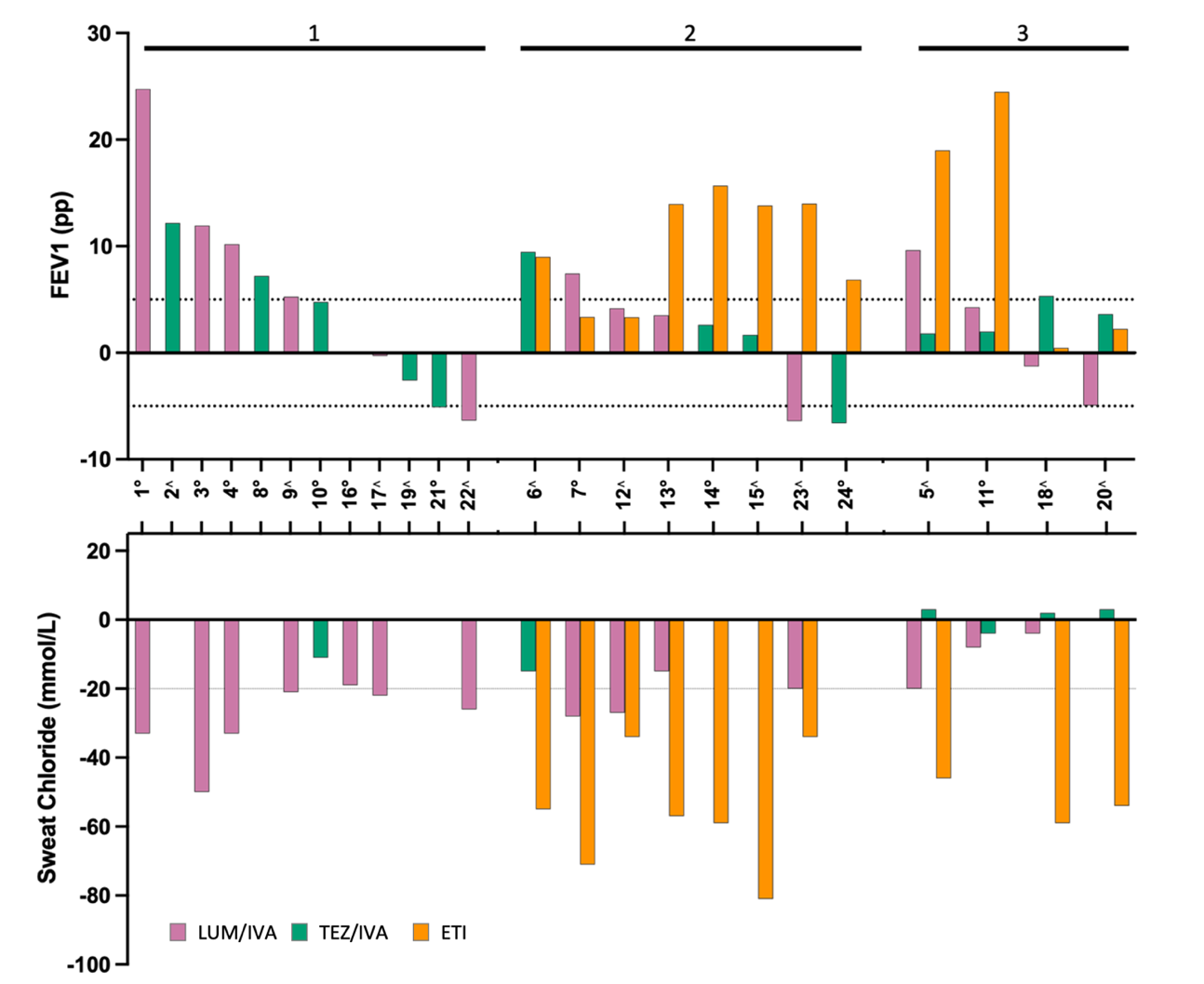

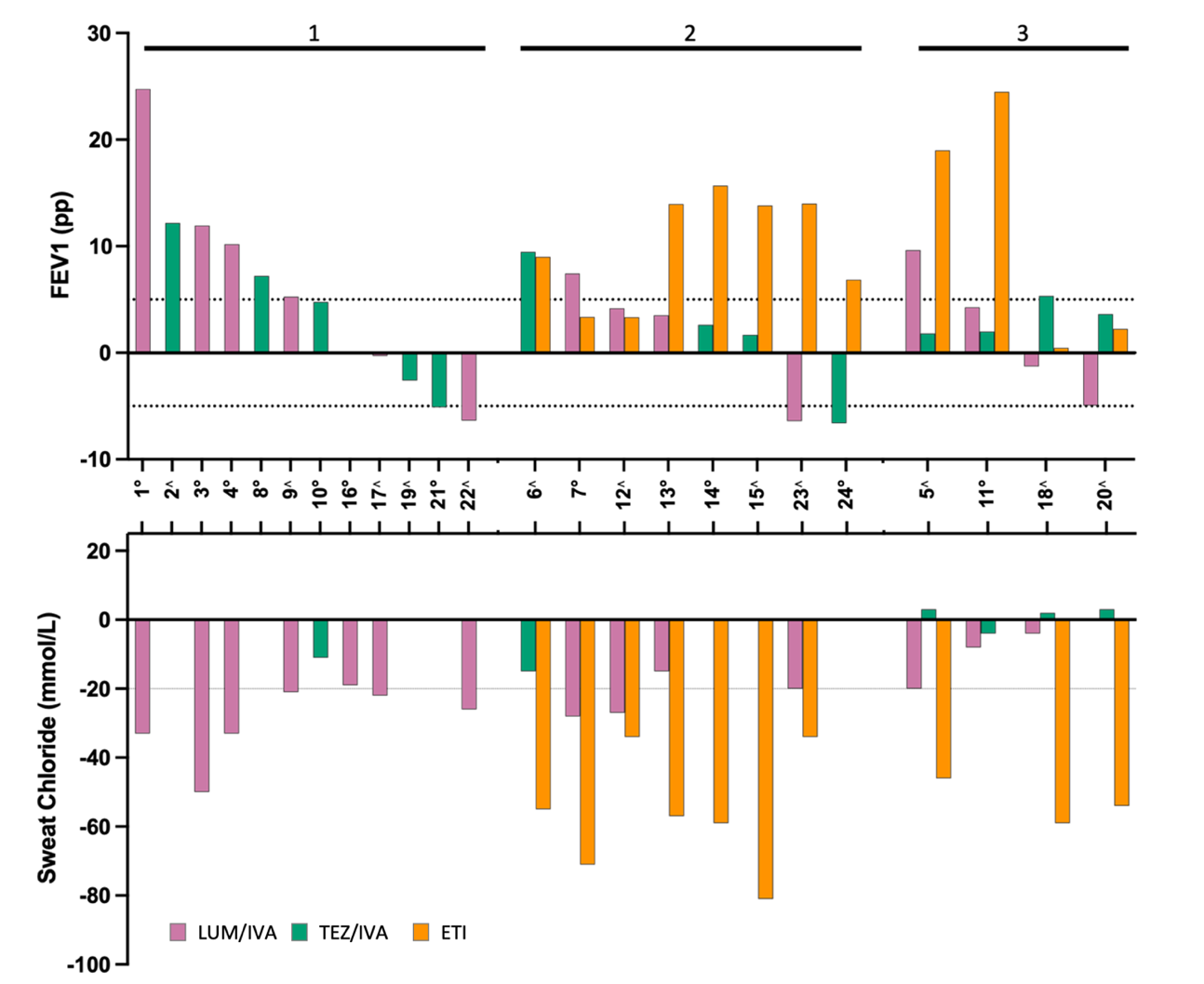

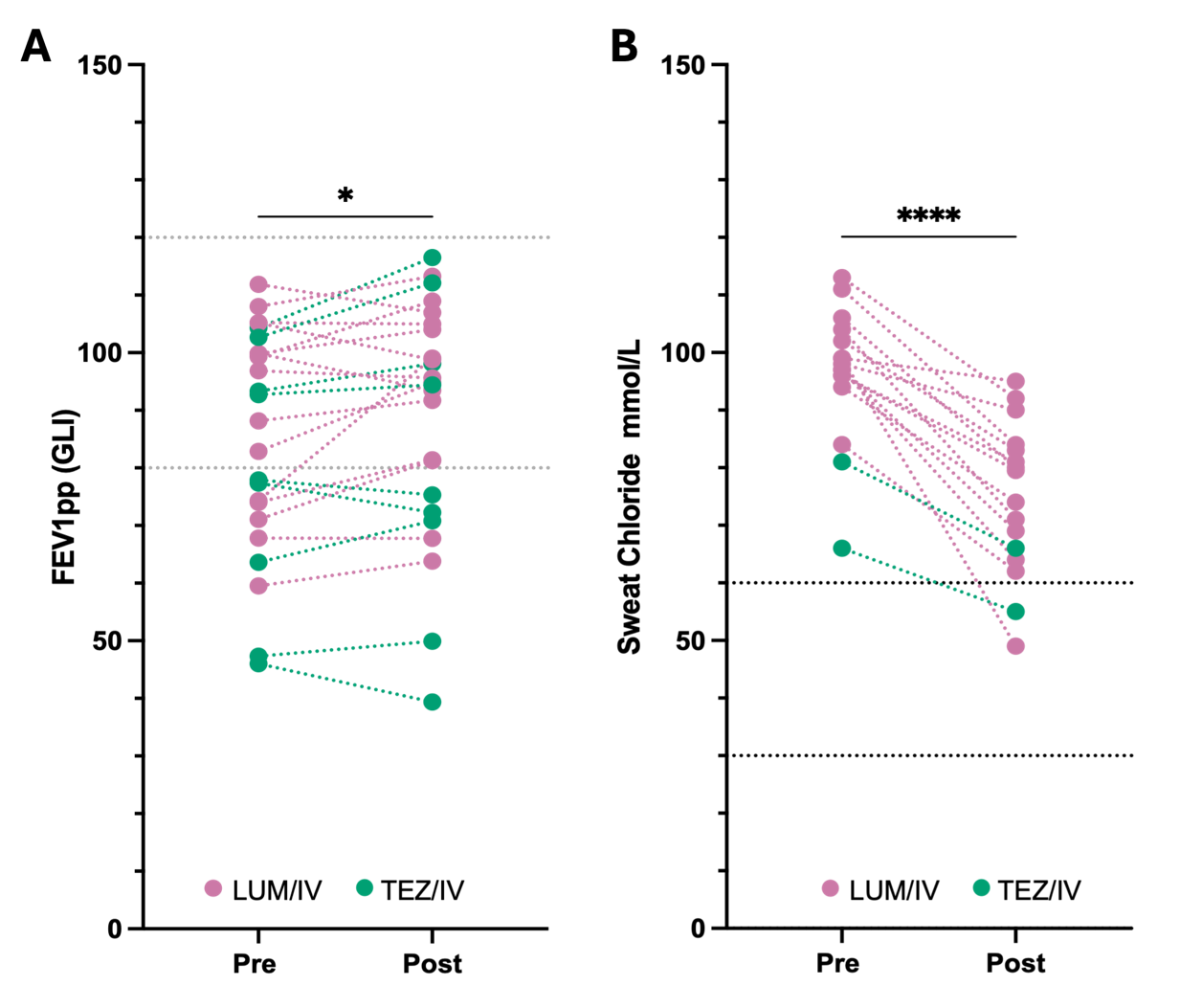


**C**


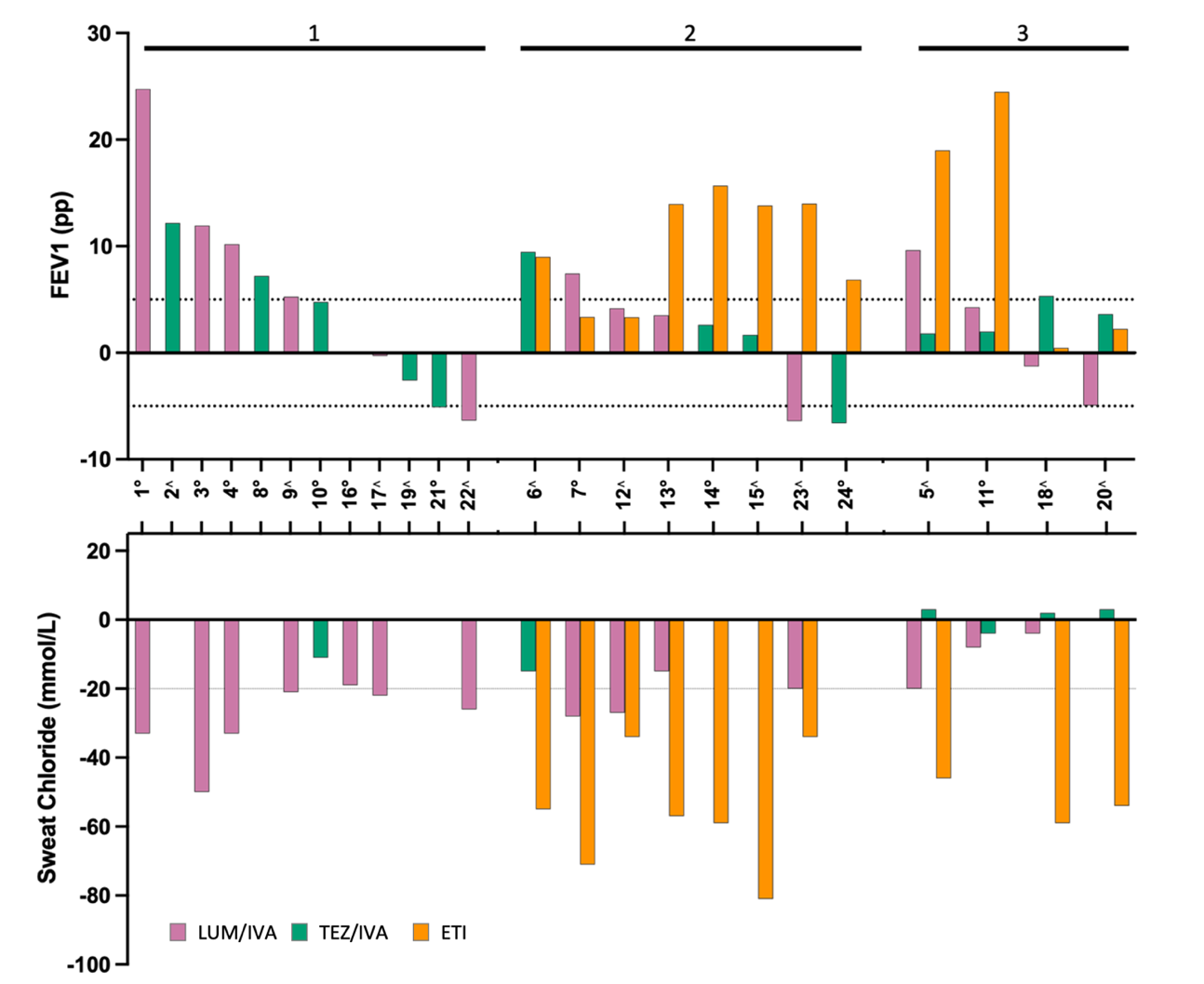


**Figure S2: Heterogenous i*n vivo* clinical response to CFTR modulators. A) Paired FEV1pp results pre and post CFTR modulator treatment. R**esults were compared with a paired t test. Each pair of dots represents an individual patient. **B) Paired sweat chloride results pre and post CFTR modulator treatment.** Results were compared with a paired t test. Each pair of dots represents an individual patient**. C)Waterfall plots illustrate the absolute change in participant outcomes after treatment with CFTR modulators.** Results are displayed with data grouped per participant. Participants are then grouped by the number of modulator treatments received (1, 2 or 3). Absolute change in FEV1pp following treatment with lumacaftor/ivacaftor (LUM/IVA), tezacaftor/ivacaftor (TEZ/IVA) or elexacaftor/ tezacaftor/ivacaftor (ETI) is displayed on the top graph with the corresponding participant’s absolute change in SC level displayed on the lower graph. Nine participants had incomplete SC data (**Table S2**). Each bar represents an individual CFTR modulator response, with significant changes (FEV1pp change of >5 percentage points; SC decrease of > 20mmol/L) indicated by dotted lines. Participants are identified using a participant de-identifier. Symbols denote participants with a baseline FEV1pp below 90 (∘) or above 90 (∧). FEV1pp: Forced Expiratory Volume in 1 second, percent predicted. SC: Sweat Chloride.


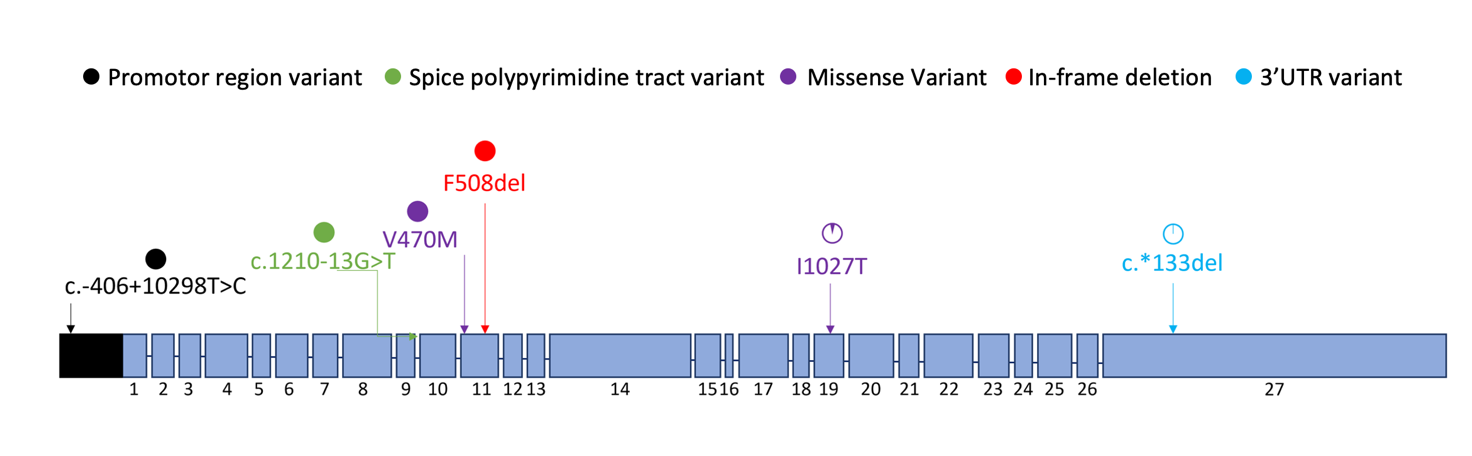

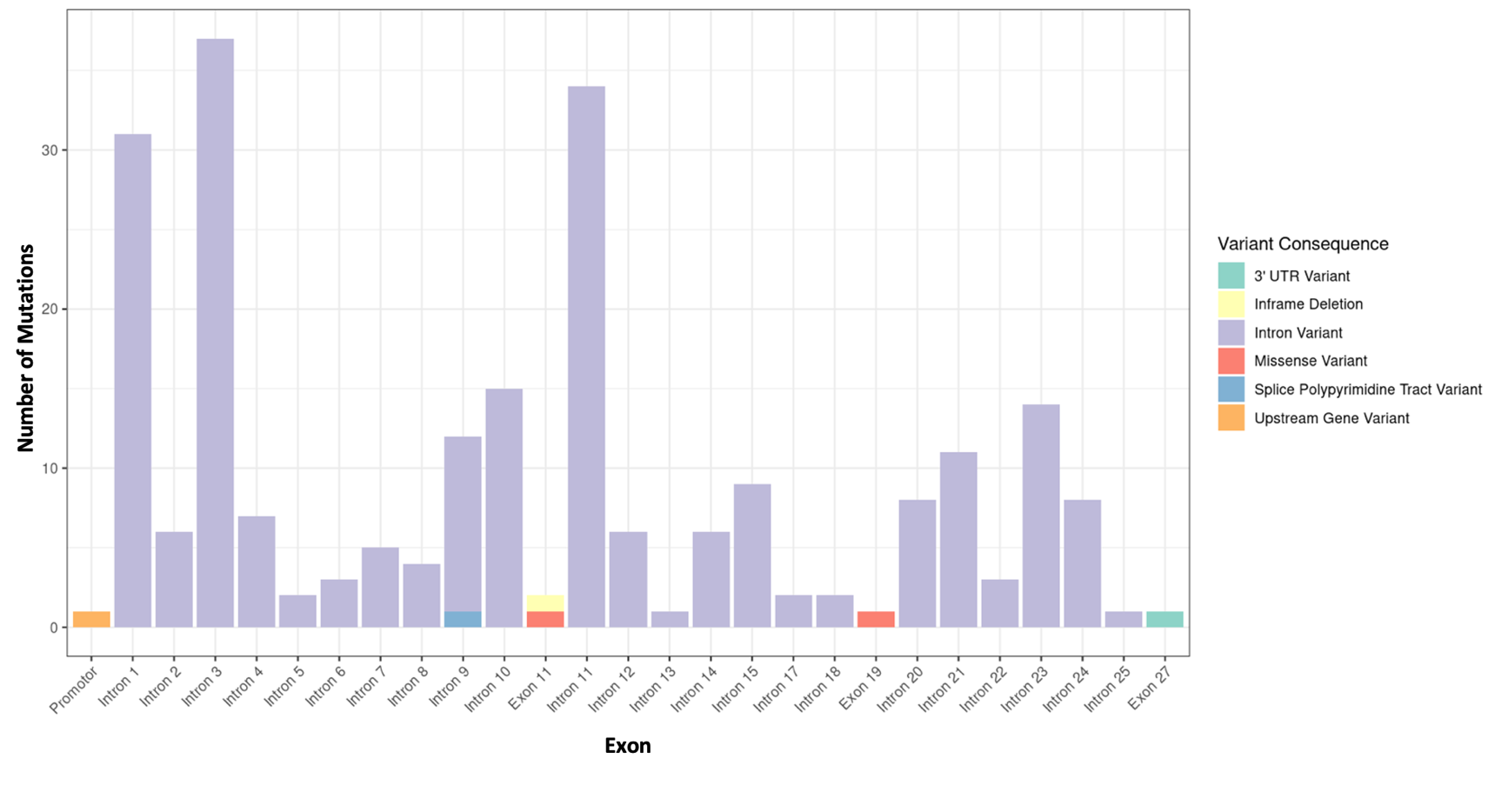


**A**

**B**

**Figure S3: Distribution and frequency of identified variants in the CFTR gene of study participants. A) Schematic of non-intronic variants’ location in the CFTR gene.** Each blue box represents a numbered exon, indicating its position within the gene. The frequency of each identified allele in the cohort is visualized with a progress circle, where each slice denotes a percentage of the total occurrences of that allele. Variants which were found to be homozygous in all individuals are represented by fully shaded circles. Different colours indicate the type of variant**. B) Histogram of the frequency and distribution of the 231 identified variants within the CFTR gene in our participants.** Each bar represents one exon or intron. Only introns and exons with a frequency greater or equal to 1 are shown. Different colours highlight the type of variant.

**
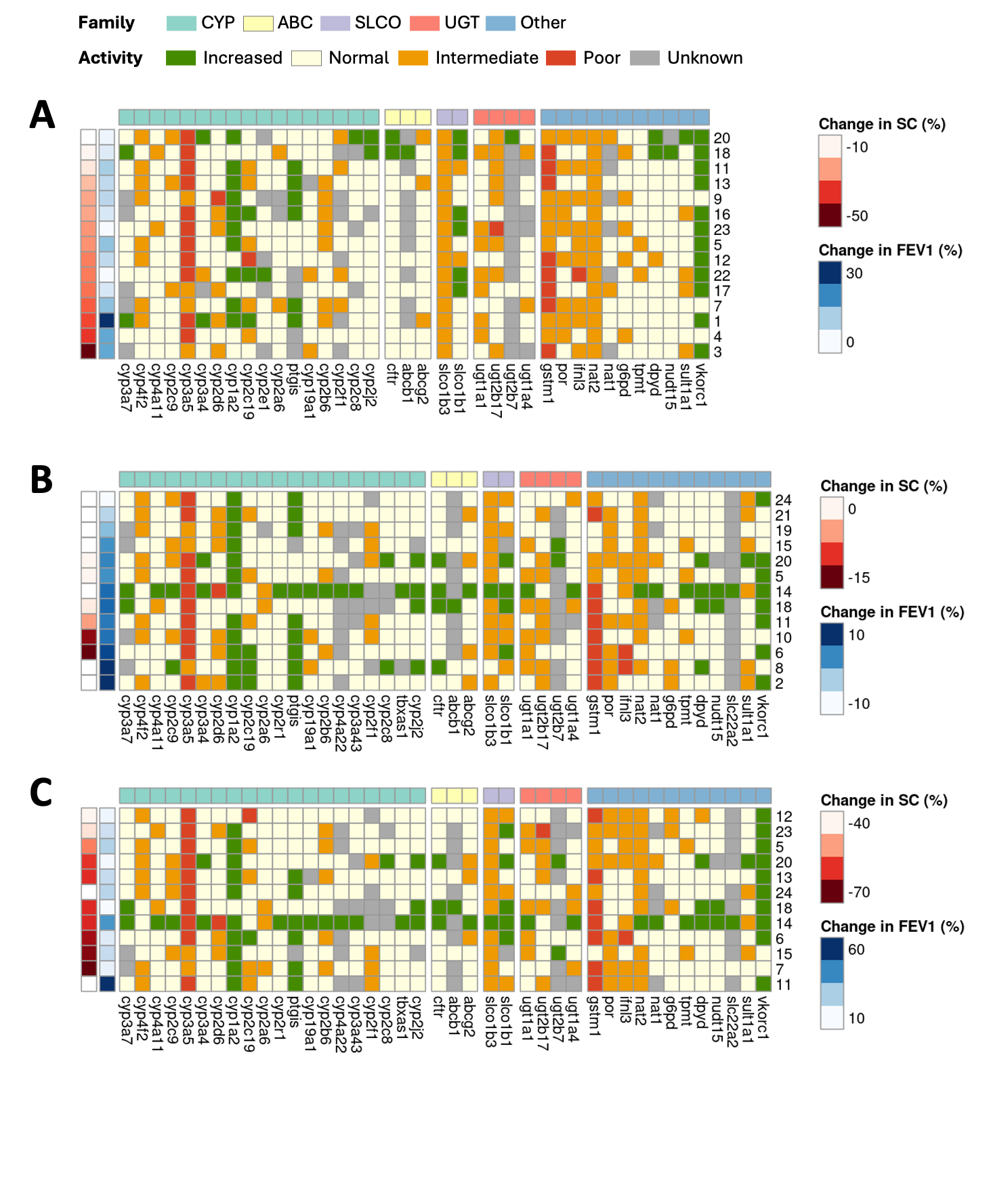
Figure S4**: **Pharmacogene profile across participants by each CFTR modulator.** Pharmacogene function following treatment with **A)** Lumacaftor/Ivacaftor, **B)** Tezacaftor/Ivacaftor and **C)** Elexacaftor/Tezacaftor/Ivacaftor. The predicted activity levels of pharmacogenes were categorised for 58 genes from the Cytochrome P450 enzymes (CYP), ATP-binding cassette transporters (ABC), solute carrier families (SLC and SLCO) and UDP-glucuronosyltransferases (UGT). Activity levels are indicated based on their Stargazer score; increased (> 2), normal (= 2), intermediate (1 -2), poor (0 - 1) and unknown (< 0). Each column contains the predicted activity of a pharmacogene. Participants were ranked primarily by their responsiveness to treatment as measured by the relative increase in SC. FEV1pp was used to further rank participants with missing SC data. Each participant (n=24) is represented once per treatment and may be included up to 3 times. Pharmacogenes are not shown when the output for all participants was “normal” or “unknown”. SC: sweat chloride. FEV1pp: forced expiratory volume in one second, percent predicted.

**Table S3**: Information about the 231 additional variants detected in the cohort.

| HGSVc name | Position | Alternate Allele | Consequence | Clinical Significance | Patient AF | GnomADg AF |
| --- | --- | --- | --- | --- | --- | --- |
|  |  |  |  |  |  |  |
| c.*133del | 7:117667232-117667233 | - | 3' UTR Variant | Likely Benign | 0.02083333 | 0.4729 |
| c.53+10377T>C | 7:117490524-117490524 | C | Intron Variant | - | 0.02083333 | 6.58E-06 |
| c.54-5282A>G | 7:117498971-117498971 | G | Intron Variant | - | 0.02083333 | - |
| c.54-2505_54-2504insAAAAAAA  AAAAAAAAAAAAAAAA | 7:117501747-117501748 | AAAAAAAAAAAAAAA  AAAAAAAAA | Intron Variant | - | 0.02083333 | - |
| c.54-2460C>A | 7:117501793-117501793 | A | Intron Variant | - | 0.02083333 | 2.79E-02 |
| c.273+4567dup | 7:117513707-117513707 | T | Intron Variant | - | 0.02083333 | 1.97E-05 |
| c.273+8143_273+8146dup | 7:117517283-117517283 | ACTT | Intron Variant | - | 0.02083333 | - |
| c.869+993C>T | 7:117537666-117537666 | T | Intron Variant | - | 0.02083333 | - |
| c.1116+433_1116+436del | 7:117540777-117540781 | - | Intron Variant | - | 0.02083333 | - |
| c.1116+435_1116+436insCCCC | 7:117540781-117540781 | CCCC | Intron Variant | - | 0.02083333 | - |
| c.1210-285_1210-276dup | 7:117548333-117548333 | GTGTGTGTGT | Intron Variant | - | 0.02083333 | 1.63E-02 |
| c.1393-4892T>C | 7:117554572-117554572 | C | Intron Variant | - | 0.02083333 | 1.51E-04 |
| c.1393-2952_1393-2951del | 7:117556510-117556512 | - | Intron Variant | - | 0.02083333 | - |
| c.1393-878_1393-875dup | 7:117558553-117558553 | TAAA | Intron Variant | - | 0.02083333 | 0.151 |
| c.1393-886_1393-875dup | 7:117558553-117558553 | TAAATAAATAAA | Intron Variant | - | 0.02083333 | 2.74E-02 |
| c.1584+6221G>A | 7:117565876-117565876 | A | Intron Variant | - | 0.02083333 | 6.58E-06 |
| c.1584+6624_1584+6631dup | 7:117566248-117566248 | ACACACAC | Intron Variant | - | 0.02083333 | 3.20E-02 |
| c.1584+11155A>G | 7:117570810-117570810 | G | Intron Variant | - | 0.02083333 | 5.26E-05 |
| c.2490+970G>A | 7:117593627-117593627 | A | Intron Variant | - | 0.02083333 | 6.58E-05 |
| c.2490+1086G>A | 7:117593743-117593743 | A | Intron Variant | - | 0.02083333 | 4.61E-05 |
| c.2619+2715T>C | 7:117597773-117597773 | C | Intron Variant | - | 0.02083333 | 1.45E-02 |
| c.2619+3341A>C | 7:117598399-117598399 | C | Intron Variant | - | 0.02083333 | 0.1556 |
| c.2620-2212A>T | 7:117600614-117600614 | T | Intron Variant | - | 0.02083333 | 0.4705 |
| c.2620-1849C>A | 7:117600977-117600977 | A | Intron Variant | - | 0.02083333 | 0.4809 |
| c.2909-413A>C | 7:117606261-117606261 | C | Intron Variant | - | 0.02083333 | 0.1509 |
| c.2988+1465del | 7:117608207-117608208 | - | Intron Variant | - | 0.02083333 | 0.1507 |
| c.3367+213_3367+214dup | 7:117612006-117612006 | AT | Intron Variant | - | 0.02083333 | 8.73E-02 |
| c.3367+215_3367+222del | 7:117612015-117612023 | - | Intron Variant | - | 0.02083333 | 2.24E-05 |
| c.3367+233_3367+234insC | 7:117612041-117612041 | C | Intron Variant | - | 0.02083333 | - |
| c.3367+265A>C | 7:117612073-117612073 | C | Intron Variant | - | 0.02083333 | - |
| c.3367+1115A>G | 7:117612923-117612923 | G | Intron Variant | - | 0.02083333 | 3.23E-02 |
| c.3468+1607C>T | 7:117616320-117616320 | T | Intron Variant | - | 0.02083333 | 0.1485 |
| c.3468+2920C>T | 7:117617633-117617633 | T | Intron Variant | - | 0.02083333 | 0.1074 |
| c.3468+3605A>G | 7:117618318-117618318 | G | Intron Variant | - | 0.02083333 | 0.4282 |
| c.3468+3762G>A | 7:117618475-117618475 | A | Intron Variant | - | 0.02083333 | 0.4815 |
| c.3468+3792_3468+3793dup | 7:117618478-117618478 | CA | Intron Variant | - | 0.02083333 | 0.2306 |
| c.3468+4244G>A | 7:117618957-117618957 | A | Intron Variant | - | 0.02083333 | 0.47 |
| c.3468+5372del | 7:117620074-117620075 | - | Intron Variant | - | 0.02083333 | 0.4678 |
| c.3469-6283C>T | 7:117621239-117621239 | T | Intron Variant | - | 0.02083333 | 0.4698 |
| c.3469-3450T>C | 7:117624072-117624072 | C | Intron Variant | - | 0.02083333 | 0.1487 |
| c.3718-5607dup | 7:117636818-117636818 | T | Intron Variant | - | 0.02083333 | 7.14E-03 |
| c.3718-5203dup | 7:117637222-117637222 | T | Intron Variant | - | 0.02083333 | 0.3954 |
| c.3873+3877G>A | 7:117646470-117646470 | A | Intron Variant | - | 0.02083333 | 0.4661 |
| c.3873+5101del | 7:117647679-117647680 | - | Intron Variant | - | 0.02083333 | 0.4388 |
| c.3874-4761_3874-4760dup | 7:117648069-117648069 | TA | Intron Variant | - | 0.02083333 | 0.1584 |
| c.3874-3564A>G | 7:117649278-117649278 | G | Intron Variant | - | 0.02083333 | 0.4316 |
| c.3874-3357G>T | 7:117649485-117649485 | T | Intron Variant | - | 0.02083333 | 0.3665 |
| c.3874-3356T>A | 7:117649486-117649486 | A | Intron Variant | - | 0.02083333 | 0.3633 |
| c.3874-3327del | 7:117649514-117649515 | - | Intron Variant | - | 0.02083333 | 0.2423 |
| c.3874-3325_3874-3323del | 7:117649516-117649519 | - | Intron Variant | - | 0.02083333 | 0.1589 |
| c.3874-3325del | 7:117649516-117649519 | TA | Intron Variant | - | 0.02083333 | 0.2291 |
| c.3874-3323del | 7:117649518-117649519 | - | Intron Variant | - | 0.02083333 | 0.116 |
| c.3874-3222G>T | 7:117649620-117649620 | T | Intron Variant | - | 0.02083333 | 0.1507 |
| c.3874-560C>T | 7:117652282-117652282 | T | Intron Variant | - | 0.02083333 | 0.1485 |
| c.3964-5607T>C | 7:117659081-117659081 | C | Intron Variant | - | 0.02083333 | 1.31E-05 |
| c.3964-4000_3964-3999dup | 7:117660666-117660666 | TG | Intron Variant | - | 0.02083333 | 0.1713 |
| c.3964-2704_3964-2702del | 7:117661983-117661986 | - | Intron Variant | - | 0.02083333 | 0.3659 |
| c.3964-2702dup | 7:117661983-117661986 | AAAA | Intron Variant | - | 0.02083333 | 3.39E-02 |
| c.3964-1832A>C | 7:117662856-117662856 | C | Intron Variant | - | 0.02083333 | 0.1502 |
| c.3964-1162_3964-1161del | 7:117663509-117663511 | - | Intron Variant | - | 0.02083333 | 1.92E-02 |
| c.3964-1091G>A | 7:117663597-117663597 | A | Intron Variant | - | 0.02083333 | 1.37E-02 |
| c.2989-81dup | 7:117610425-117610425 | A | Intron Variant | Benign | 0.02083333 | 2.04E-02 |
| c.4136+237C>T | 7:117665097-117665097 | T | Intron Variant | Benign | 0.02083333 | 0.1503 |
| c.54-8086A>G | 7:117496167-117496167 | G | Intron Variant | Likely Benign | 0.02083333 | 1.45E-04 |
| c.273+1108T>G | 7:117510250-117510250 | G | Intron Variant | - | 0.04166667 | 9.85E-05 |
| c.489+975del | 7:117532077-117532078 | - | Intron Variant | - | 0.04166667 | 2.48E-03 |
| c.1210-287_1210-276dup | 7:117548333-117548333 | GTGTGTGTGTGT | Intron Variant | - | 0.04166667 | 9.86E-03 |
| c.1584+5097G>A | 7:117564752-117564752 | A | Intron Variant | - | 0.04166667 | 1.58E-04 |
| c.1584+6618_1584+6631dup | 7:117566248-117566248 | ACACACACACACAC | Intron Variant | - | 0.04166667 | 1.79E-03 |
| c.1584+6620_1584+6631dup | 7:117566248-117566248 | ACACACACACAC | Intron Variant | - | 0.04166667 | 4.97E-03 |
| c.3368-589dup | 7:117613999-117613999 | T | Intron Variant | - | 0.04166667 | 5.43E-02 |
| c.1393-2843G>A | 7:117556621-117556621 | A | Intron Variant | - | 0.0625 | 9.22E-05 |
| c.3080T>C | 7:117610610-117610610 | C | Missense Variant | Benign/Likely Benign | 0.0625 | 3.81E-04 |
| c.1584+6626_1584+6631dup | 7:117566248-117566248 | ACACAC | Intron Variant | - | 0.104166667 | 7.82E-02 |
| c.3367+242_3367+245dup | 7:117612023-117612023 | TATA | Intron Variant | - | 0.20833333 | 0.1018 |
| c.1210-277_1210-276dup | 7:117548333-117548333 | GT | Intron Variant | - | 0.45833333 | 0.1934 |
| c.1210-289_1210-276dup | 7:117548333-117548333 | GTGTGTGTGTGTGT | Intron Variant | - | 0.47916667 | 7.21E-02 |
| c.3367+244_3367+245dup | 7:117612023-117612023 | TA | Intron Variant | - | 0.47916667 | 0.2038 |
| c.1584+6622_1584+6631dup | 7:117566248-117566248 | ACACACACAC | Intron Variant | - | 0.79166667 | 2.36E-02 |
| c.54-2505del | 7:117501747-117501748 | - | Intron Variant | - | 0.89583333 | 0.2199 |
| c.2909-650G>A | 7:117606024-117606024 | A | Intron Variant | - | 0.9375 | 1.63E-02 |
| c.3718-4938A>T | 7:117637500-117637500 | T | Intron Variant | - | 0.9375 | 1.23E-02 |
| c.3874-1764A>G | 7:117651078-117651078 | G | Intron Variant | - | 0.9375 | 8.41E-02 |
| c.3964-5308G>A | 7:117659380-117659380 | A | Intron Variant | - | 0.9375 | 1.14E-02 |
| c.54-2467A>C | 7:117501786-117501786 | C | Intron Variant | - | 0.95833333 | 0.2694 |
| c.1393-882_1393-875dup | 7:117558553-117558553 | TAAATAAA | Intron Variant | - | 0.95833333 | 0.1746 |
| c.1393-2928_1393-2927del | 7:117556512-117556514 | - | Intron Variant | - | 0.97916667 | 0.1689 |
| c.2619+3267T>G | 7:117598325-117598325 | G | Intron Variant | - | 0.97916667 | 0.5392 |
| c.2619+3333T>G | 7:117598391-117598391 | G | Intron Variant | - | 0.97916667 | 0.5447 |
| c.53+2253T>G | 7:117482400-117482400 | G | Intron Variant | - | 1 | 7.57E-02 |
| c.53+2664G>A | 7:117482811-117482811 | A | Intron Variant | - | 1 | 7.49E-02 |
| c.53+6768_53+6771dup | 7:117486896-117486896 | GAGA | Intron Variant | - | 1 | 0.1392 |
| c.53+7538A>C | 7:117487685-117487685 | C | Intron Variant | - | 1 | 0.2507 |
| c.53+8741A>C | 7:117488888-117488888 | C | Intron Variant | - | 1 | 0.548 |
| c.53+9203T>C | 7:117489350-117489350 | C | Intron Variant | - | 1 | 0.5504 |
| c.53+10442G>C | 7:117490589-117490589 | C | Intron Variant | - | 1 | 0.2296 |
| c.53+10983C>T | 7:117491130-117491130 | T | Intron Variant | - | 1 | 0.1169 |
| c.53+11897T>A | 7:117492044-117492044 | A | Intron Variant | - | 1 | 0.2304 |
| c.53+11978A>G | 7:117492125-117492125 | G | Intron Variant | - | 1 | 0.2357 |
| c.54-9813del | 7:117494438-117494439 | - | Intron Variant | - | 1 | 0.5551 |
| c.54-9790C>G | 7:117494463-117494463 | G | Intron Variant | - | 1 | 0.2509 |
| c.54-9673G>A | 7:117494580-117494580 | A | Intron Variant | - | 1 | 0.235 |
| c.54-9282G>C | 7:117494971-117494971 | C | Intron Variant | - | 1 | 0.2304 |
| c.54-9095C>T | 7:117495158-117495158 | T | Intron Variant | - | 1 | 6.11E-02 |
| c.54-8089dup | 7:117496156-117496156 | T | Intron Variant | - | 1 | 0.1272 |
| c.54-7449T>G | 7:117496804-117496804 | G | Intron Variant | - | 1 | 0.2497 |
| c.54-6907A>C | 7:117497346-117497346 | C | Intron Variant | - | 1 | 0.2506 |
| c.54-5360A>G | 7:117498893-117498893 | G | Intron Variant | - | 1 | 0.2497 |
| c.54-4796_54-4791dup | 7:117499429-117499429 | TGTGTG | Intron Variant | - | 1 | 0.1631 |
| c.54-4212C>T | 7:117500041-117500041 | T | Intron Variant | - | 1 | 0.2289 |
| c.54-3963_54-3950del | 7:117500278-117500292 | - | Intron Variant | - | 1 | 0.2147 |
| c.54-705C>G | 7:117503548-117503548 | G | Intron Variant | - | 1 | 0.2505 |
| c.54-589A>G | 7:117503664-117503664 | G | Intron Variant | - | 1 | 6.78E-02 |
| c.164+410del | 7:117504756-117504757 | - | Intron Variant | - | 1 | 8.72E-02 |
| c.164+1976T>C | 7:117506339-117506339 | C | Intron Variant | - | 1 | 0.249 |
| c.164+2031T>C | 7:117506394-117506394 | C | Intron Variant | - | 1 | 0.2505 |
| c.165-1883T>C | 7:117507151-117507151 | C | Intron Variant | - | 1 | 0.2357 |
| c.165-1588C>G | 7:117507446-117507446 | G | Intron Variant | - | 1 | 0.5676 |
| c.165-1541C>T | 7:117507493-117507493 | T | Intron Variant | - | 1 | 0.2301 |
| c.273+879C>A | 7:117510021-117510021 | A | Intron Variant | - | 1 | 0.2356 |
| c.273+922G>C | 7:117510064-117510064 | C | Intron Variant | - | 1 | 0.2502 |
| c.273+933C>T | 7:117510075-117510075 | T | Intron Variant | - | 1 | 0.2504 |
| c.273+2632A>C | 7:117511774-117511774 | C | Intron Variant | - | 1 | 0.5574 |
| c.273+3512del | 7:117512633-117512634 | - | Intron Variant | - | 1 | 0.4709 |
| c.273+4309dup | 7:117513437-117513437 | A | Intron Variant | - | 1 | 0.5303 |
| c.273+4963G>A | 7:117514105-117514105 | A | Intron Variant | - | 1 | 0.2349 |
| c.273+5802A>C | 7:117514944-117514944 | C | Intron Variant | - | 1 | 0.236 |
| c.273+6647A>G | 7:117515789-117515789 | G | Intron Variant | - | 1 | 0.251 |
| c.273+7727T>G | 7:117516869-117516869 | G | Intron Variant | - | 1 | 0.2302 |
| c.273+8685_273+8686dup | 7:117517826-117517826 | AT | Intron Variant | - | 1 | 0.2514 |
| c.273+9446A>G | 7:117518588-117518588 | G | Intron Variant | - | 1 | 0.4276 |
| c.274-9965A>G | 7:117520934-117520934 | G | Intron Variant | - | 1 | 0.235 |
| c.274-9300T>G | 7:117521599-117521599 | G | Intron Variant | - | 1 | 0.5567 |
| c.274-7999A>G | 7:117522900-117522900 | G | Intron Variant | - | 1 | 0.2301 |
| c.274-7920G>C | 7:117522979-117522979 | C | Intron Variant | - | 1 | 7.51E-02 |
| c.274-7868T>A | 7:117523031-117523031 | A | Intron Variant | - | 1 | 7.51E-02 |
| c.274-7505dup | 7:117523381-117523381 | T | Intron Variant | - | 1 | 0.2371 |
| c.274-7337A>G | 7:117523562-117523562 | G | Intron Variant | - | 1 | 7.52E-02 |
| c.274-7279T>G | 7:117523620-117523620 | G | Intron Variant | - | 1 | 0.2505 |
| c.274-6183G>A | 7:117524716-117524716 | A | Intron Variant | - | 1 | 7.43E-02 |
| c.274-6090T>C | 7:117524809-117524809 | C | Intron Variant | - | 1 | 0.249 |
| c.274-5806A>G | 7:117525093-117525093 | G | Intron Variant | - | 1 | 0.2516 |
| c.274-4888T>C | 7:117526011-117526011 | C | Intron Variant | - | 1 | 0.5395 |
| c.274-4549T>C | 7:117526350-117526350 | C | Intron Variant | - | 1 | 0.2101 |
| c.274-2903T>C | 7:117527996-117527996 | C | Intron Variant | - | 1 | 3.46E-04 |
| c.274-2470T>C | 7:117528429-117528429 | C | Intron Variant | - | 1 | 6.00E-05 |
| c.274-2197G>A | 7:117528702-117528702 | A | Intron Variant | - | 1 | 2.88E-04 |
| c.274-1802G>C | 7:117529097-117529097 | C | Intron Variant | - | 1 | 1.40E-02 |
| c.274-1682A>G | 7:117529217-117529217 | G | Intron Variant | - | 1 | 0.1868 |
| c.274-1367del | 7:117529513-117529514 | - | Intron Variant | - | 1 | 0.3075 |
| c.274-1352T>A | 7:117529547-117529547 | A | Intron Variant | - | 1 | 0.2275 |
| c.274-848G>A | 7:117530051-117530051 | A | Intron Variant | - | 1 | 1.95E-02 |
| c.489+586T>C | 7:117531700-117531700 | C | Intron Variant | - | 1 | 0.2302 |
| c.489+1089G>A | 7:117532203-117532203 | A | Intron Variant | - | 1 | 0.2494 |
| c.489+1101T>A | 7:117532215-117532215 | A | Intron Variant | - | 1 | 0.556 |
| c.490-1547C>T | 7:117532729-117532729 | T | Intron Variant | - | 1 | 0.2503 |
| c.490-1100T>C | 7:117533176-117533176 | C | Intron Variant | - | 1 | 0.5574 |
| c.490-875T>G | 7:117533401-117533401 | G | Intron Variant | - | 1 | 0.2355 |
| c.579+385T>A | 7:117534750-117534750 | A | Intron Variant | - | 1 | 0.2357 |
| c.744-496A>G | 7:117536052-117536052 | G | Intron Variant | - | 1 | 0.2349 |
| c.744-354A>G | 7:117536194-117536194 | G | Intron Variant | - | 1 | 0.235 |
| c.744-9_744-6del | 7:117536514-117536518 | - | Intron Variant | - | 1 | 0.2322 |
| c.869+731dup | 7:117537399-117537399 | T | Intron Variant | - | 1 | 0.2303 |
| c.869+1481A>G | 7:117538154-117538154 | G | Intron Variant | - | 1 | 0.557 |
| c.870-1345del | 7:117538753-117538754 | - | Intron Variant | - | 1 | 0.5664 |
| c.1116+612C>T | 7:117540958-117540958 | T | Intron Variant | - | 1 | 0.1168 |
| c.1117-358del | 7:117541650-117541651 | - | Intron Variant | - | 1 | 0.556 |
| c.1209+2354G>A | 7:117544462-117544462 | A | Intron Variant | - | 1 | 0.5562 |
| c.1209+2909T>C | 7:117545017-117545017 | C | Intron Variant | - | 1 | 0.557 |
| c.1210-2636_1210-2632dup | 7:117545984-117545984 | ATTTT | Intron Variant | - | 1 | 0.2344 |
| c.1210-1684G>A | 7:117546957-117546957 | A | Intron Variant | - | 1 | 7.43E-02 |
| c.1210-1234T>C | 7:117547407-117547407 | C | Intron Variant | - | 1 | 0.2421 |
| c.1210-1036T>C | 7:117547605-117547605 | C | Intron Variant | - | 1 | 0.2414 |
| c.1210-873G>A | 7:117547768-117547768 | A | Intron Variant | - | 1 | 0.2559 |
| c.1392+1721T>C | 7:117550544-117550544 | C | Intron Variant | - | 1 | 0.2558 |
| c.1392+2119T>G | 7:117550942-117550942 | G | Intron Variant | - | 1 | 0.2414 |
| c.1392+2406G>A | 7:117551229-117551229 | A | Intron Variant | - | 1 | 0.556 |
| c.1392+2942T>C | 7:117551765-117551765 | C | Intron Variant | - | 1 | 0.2421 |
| c.1393-3961T>C | 7:117555503-117555503 | C | Intron Variant | - | 1 | 7.50E-02 |
| c.1393-1912_1393-1899del | 7:117557545-117557559 | - | Intron Variant | - | 1 | 0.2415 |
| c.1584+772G>C | 7:117560427-117560427 | C | Intron Variant | - | 1 | 0.2568 |
| c.1584+801G>T | 7:117560456-117560456 | T | Intron Variant | - | 1 | 0.2421 |
| c.1584+1190C>T | 7:117560845-117560845 | T | Intron Variant | - | 1 | 0.1238 |
| c.1584+2775C>G | 7:117562430-117562430 | G | Intron Variant | - | 1 | 0.2367 |
| c.1584+8151C>A | 7:117567806-117567806 | A | Intron Variant | - | 1 | 0.4109 |
| c.1584+12144T>C | 7:117571799-117571799 | C | Intron Variant | - | 1 | 0.4209 |
| c.1584+12338dup | 7:117571986-117571986 | T | Intron Variant | - | 1 | 0.4121 |
| c.1584+13014T>C | 7:117572669-117572669 | C | Intron Variant | - | 1 | 0.5525 |
| c.1585-13420G>C | 7:117574319-117574319 | C | Intron Variant | - | 1 | 0.2054 |
| c.1585-13322T>G | 7:117574417-117574417 | G | Intron Variant | - | 1 | 7.13E-02 |
| c.1585-10072C>T | 7:117577667-117577667 | T | Intron Variant | - | 1 | 0.5456 |
| c.1585-10068C>T | 7:117577671-117577671 | T | Intron Variant | - | 1 | 0.1953 |
| c.1585-9806T>C | 7:117577933-117577933 | C | Intron Variant | - | 1 | 6.30E-02 |
| c.1585-9634T>A | 7:117578105-117578105 | A | Intron Variant | - | 1 | 0.3819 |
| c.1585-7390A>G | 7:117580349-117580349 | G | Intron Variant | - | 1 | 0.2065 |
| c.1585-6932A>G | 7:117580807-117580807 | G | Intron Variant | - | 1 | 0.383 |
| c.1585-6787G>A | 7:117580952-117580952 | A | Intron Variant | - | 1 | 0.3825 |
| c.1585-4351T>C | 7:117583388-117583388 | C | Intron Variant | - | 1 | 1 |
| c.1585-4349G>C | 7:117583390-117583390 | C | Intron Variant | - | 1 | 1 |
| c.1585-4203A>G | 7:117583536-117583536 | G | Intron Variant | - | 1 | 0.5444 |
| c.1585-4086del | 7:117583642-117583643 | - | Intron Variant | - | 1 | 0.546 |
| c.1585-4029G>A | 7:117583710-117583710 | A | Intron Variant | - | 1 | 0.3841 |
| c.1585-3353G>A | 7:117584386-117584386 | A | Intron Variant | - | 1 | 0.9964 |
| c.1585-3069C>A | 7:117584670-117584670 | A | Intron Variant | - | 1 | 0.5438 |
| c.1585-2813T>G | 7:117584926-117584926 | G | Intron Variant | - | 1 | 8.34E-02 |
| c.1680-1240G>A | 7:117589113-117589113 | A | Intron Variant | - | 1 | 0.544 |
| c.1680-1071A>G | 7:117589282-117589282 | G | Intron Variant | - | 1 | 0.544 |
| c.1680-588A>G | 7:117589765-117589765 | G | Intron Variant | - | 1 | 0.5438 |
| c.1680-570G>T | 7:117589783-117589783 | T | Intron Variant | - | 1 | 0.544 |
| c.1680-414C>T | 7:117589939-117589939 | T | Intron Variant | - | 1 | 0.5443 |
| c.1766+653C>T | 7:117591092-117591092 | T | Intron Variant | - | 1 | 0.9695 |
| c.2490+983G>C | 7:117593640-117593640 | C | Intron Variant | - | 1 | 0.5434 |
| c.2491-808G>A | 7:117594122-117594122 | A | Intron Variant | - | 1 | 0.5443 |
| c.2491-586T>A | 7:117594344-117594344 | A | Intron Variant | - | 1 | 0.1052 |
| c.2491-447A>G | 7:117594483-117594483 | G | Intron Variant | - | 1 | 0.544 |
| c.2619+774A>G | 7:117595832-117595832 | G | Intron Variant | - | 1 | 0.5444 |
| c.2619+1125dup | 7:117596181-117596181 | C | Intron Variant | - | 1 | 1 |
| c.2619+1154T>C | 7:117596212-117596212 | C | Intron Variant | - | 1 | 0.5453 |
| c.3468+1532dup | 7:117616235-117616235 | T | Intron Variant | - | 1 | 0.9927 |
| c.3469-4159G>C | 7:117623363-117623363 | C | Intron Variant | - | 1 | 0.9943 |
| c.3873+4830T>C | 7:117647423-117647423 | C | Intron Variant | - | 1 | 0.9954 |
| c.580-159G>A | 7:117535089-117535089 | A | Intron Variant | Benign | 1 | 0.1167 |
| c.869+11C>T | 7:117536684-117536684 | T | Intron Variant | Benign | 1 | 7.43E-02 |
| c.1392+395G>A | 7:117549218-117549218 | A | Intron Variant | Benign | 1 | 0.2413 |
| c.1393-61A>G | 7:117559403-117559403 | G | Intron Variant | Benign | 1 | 0.2579 |
| c.1585-9218G>A | 7:117578521-117578521 | A | Intron Variant | Benign | 1 | 0.546 |
| c.1680-870T>A | 7:117589483-117589483 | A | Intron Variant | Benign | 1 | 0.544 |
| c.274-179G>A | 7:117530720-117530720 | A | Intron Variant | Benign/Likely Benign | 1 | 0.2515 |
| c.1408G>A | 7:117559479-117559479 | A | Missense Variant | Benign | 1 | 0.557 |
| c.1210-13G>T | 7:117548628-117548628 | T | Splice Polypyrimidine Tract Variant, Intron Variant | Benign/Likely Benign | 1 | 9.47E-02 |

GnomADg AF is the frequency of the variant in GnomAD genomes combined populations.

Clinical significance is based on ClinVar predictions.


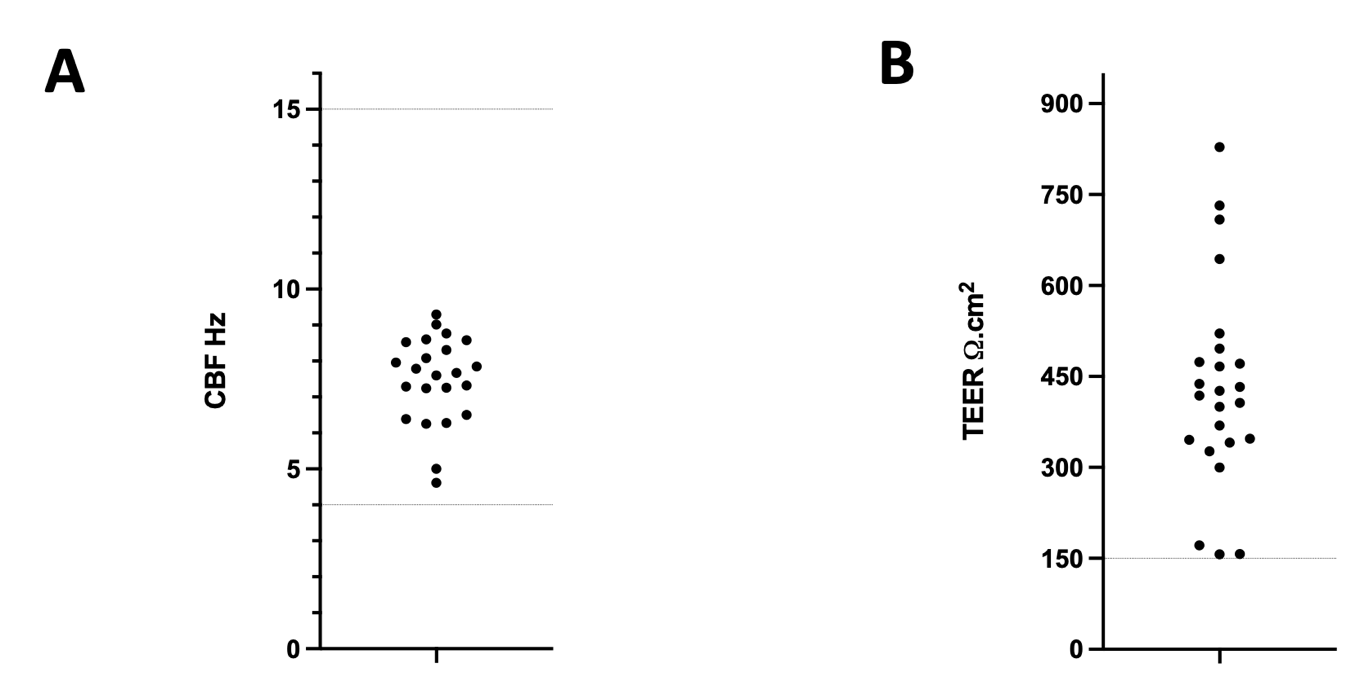


**Figure S5: Validation of *in vitro* differentiated-HNEC cultures prior to electrophysiological assessment. A) Ciliary beat frequency** **(CBF).** Baseline measurements of mature fully differentiated human nasal epithelial cell (HNEC) cultures prior to treatment with CFTR correctors. Each dot represents one individual (n = 23 due to failed data acquisition for one participant). Mean CBF 7.49Hz (95% CI 6.96 – 8.01). Dotted lines represent the upper and lower limits of normal values[6]. Data are represented as the mean of at least four replicate differentiated-HNEC cultures per participant. An average of six fields of view (FOV) were measured per differentiated-HNEC culture.  **B) Trans epithelial electrical resistance (TEER) measurements.** TEER was measured during electrophysiological analysis of short circuit current in an Ussing chamber. Each dot represents one individual. Mean transepithelial electrical resistance (TEER) 432Ω.cm^2^ (95% CI 361 – 504). Data are represented as the mean of at least n = 6 replicate differentiated-HNEC cultures per participant. The minimal accepted value of 150Ω.cm2 is shown with a dotted line[7].

6. Nikolaizik W, Hahn J, Bauck M, et al. Comparison of ciliary beat frequencies at different temperatures in young adults. ERJ Open Res; 2020

7. Wong SL, Awatade NT, Astore MA, *et al.* Molecular Dynamics and Theratyping in Airway and Gut Organoids Reveal R352Q-CFTR Conductance Defect. *Am J Respir Cell Mol Biol*; 2022


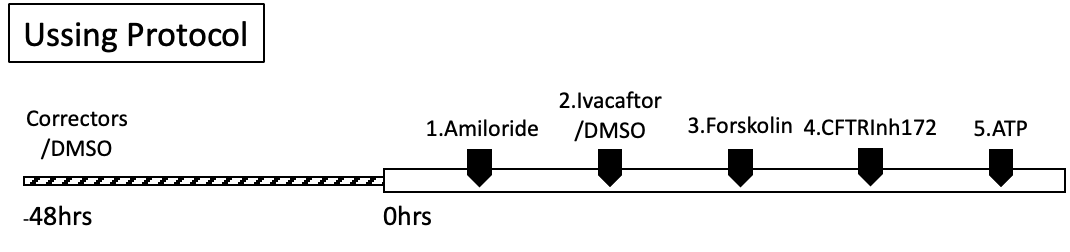


**Figure S6: Representative wild type (WT) Ussing trace.** Changes in short-circuit current (ΔIsc) in differentiated human nasal epithelial cell (HNEC) culture from WT reference (as reported in Wong et al. 2022[7]). Functional CFTR expression was measured by sequentially adding 100 μM apical amiloride (1. Amiloride), 0.01% apical DMSO vehicle control, followed by 10 μM basal forskolin (3. Forskolin), 30 μM apical CFTR inhibitor (4. CFTRinh172), and 100 μM apical ATP (5. ATP). The basolateral-to-apical chloride gradient was used to measure functional CFTR activity

7. Wong SL, Awatade NT, Astore MA, *et al.* Molecular Dynamics and Theratyping in Airway and Gut Organoids Reveal R352Q-CFTR Conductance Defect. *Am J Respir Cell Mol Biol*; 2022

**Table S4: Stats results for primary analysis of multiple linear regression for the first CFTR modulator**

| **Change in FEV1pp** | **Est** | **SE** | **DF** | **95% CI** |
| --- | --- | --- | --- | --- |
| Baseline FEV1 ≥ 90pp | 0.203 | 0.129 | 18 | -0.068, 0.473 |
| Baseline FEV1 < 90pp | -1.398 | 0.279 | 18 | -1.985, -0.811 |

**
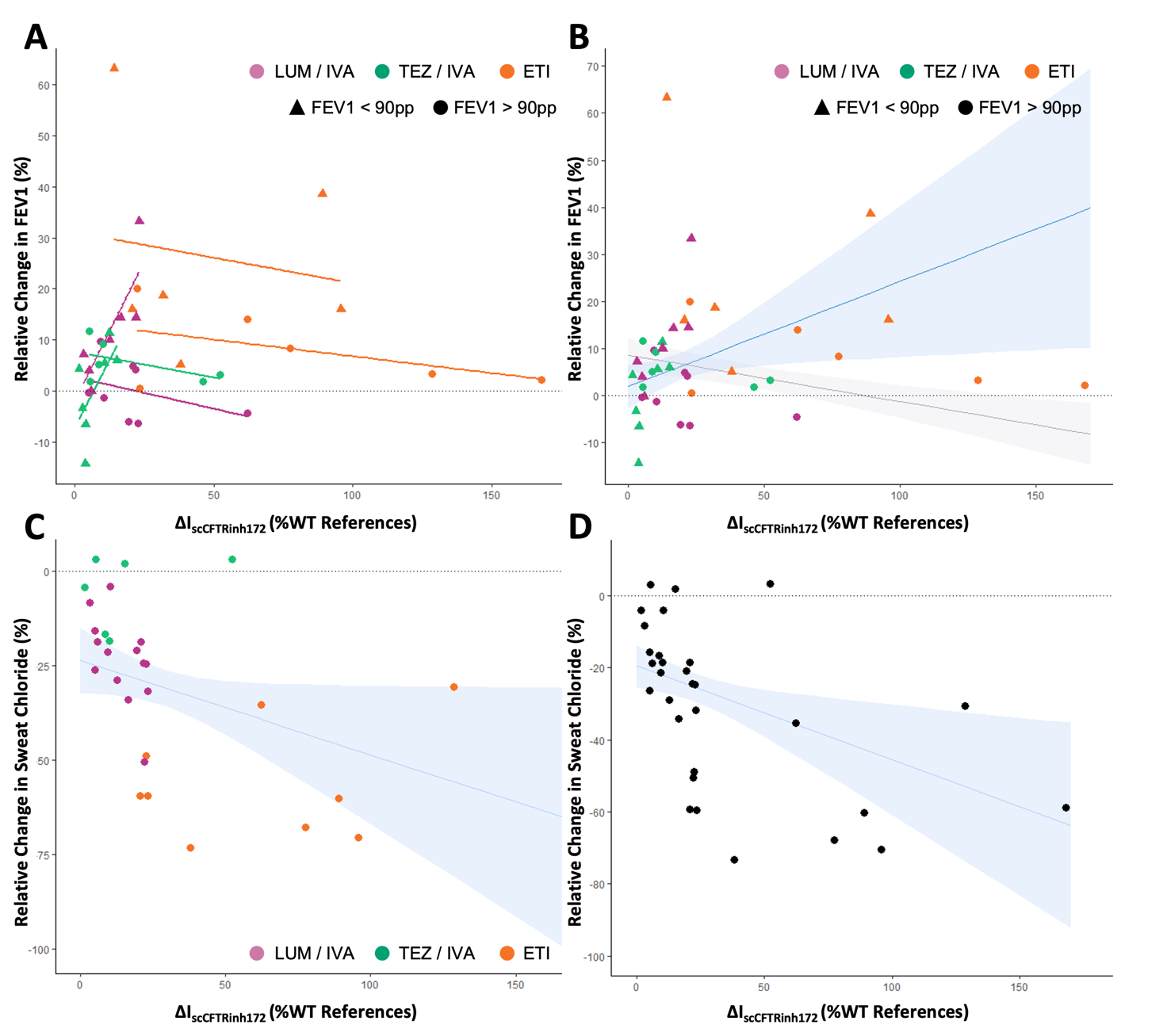
Figure S7: Exploring the relationship between *in vitro* functional responses to CFTR modulators by each CFTR modulator treatment**​**and sensitivity analysis.** **A) Scatterplot of FEV1pp vs. change in *in vitro* CFTR activity (ΔIsc) in differentiated-HNEC cultures.** Relative change in FEV1pp is plotted against ΔIsc in response to CFTR Inh172, as a percentage of the wild type reference response. Each point (dot/triangle) represents a single comparison (n = 40). Data are analysed using generalised estimating equations with a variance components covariance structure to account for repeated measures.  Separate regression lines are shown for participants with baseline FEV1pp above and below 90 and for each CFTR modulator. The colour of each point and regression line represents the specific CFTR modulator treatment administered. **B) Scatterplot of FEV1pp vs. change in *in vitro* CFTR activity (ΔIsc) in differentiated-HNEC cultures following removal of two outliers identified during sensitivity analysis of the statistical model.** Plots of relative change in FEV1pp vs ΔIsc in response to CFTRinh172 treatment. The shaded area indicates the 95% confidence interval (CI). Each point (dot/triangle) represents a single comparison (n = 38). **C) SC vs. CFTR activity (ΔIsc) in differentiated-HNEC cultures.** Plots of change in SC vs ΔIsc in response to CFTRinh172 treatment. The shaded area indicates the 95% confidence interval (CI). Each point represents a single comparison (n = 30 due to missing SC data for nine participants (**Table S2**)). The colour of each point represents the specific CFTR modulator treatment administered.  **D)** **Scatterplot of SC vs. change in *in vitro* CFTR activity (ΔIsc) in differentiated-HNEC cultures following removal of CFTR modulator treatment variable from the statistical model.** Scatterplot of relative change in SC against ΔIsc in response to CFTR Inh172, with individual comparisons represented by single dots (n=30). Data were analysed using generalised estimating equations with a variance components covariance structure to account for repeated measures. Shaded area shows 95% CI of the regression line. FEV1pp: Forced Expiratory Volume in 1 second, percent predicted. SC: Sweat Chloride.

**Table S5: Stats results for Exploratory analysis of subsequent modulators**

Analysis of 'Wald statistic' Tables

Model 1: Change in FEV1 ~ Age + Gender + Baseline FEV1 group * change in short circuit current(ΔIsc) in differentiated-HNEC cultures + CFTR modulator * ΔIsc in differentiated-HNEC cultures

Model 2: Change in FEV1 ~ Age + Gender + Baseline FEV1 group + ΔIsc in differentiated-HNEC cultures + CFTR modulator

**A: Comparison of statistical model with and without the in vitro test including sensitivity analysis**

| **Change in FEV1** | **DF** | **X^2^** | **P(>\|Chi\|)** |  |
| --- | --- | --- | --- | --- |
| All data | 4 | 14.386 | 0.006 | ** |
| Outliers removed  (identified by checking residuals) | 4 | 37.014 | <0.001 | *** |

**B: Analysis of Model 1 including sensitivity analysis**

|  |  |  |  |  | **Outliers Removed** | | |
| --- | --- | --- | --- | --- | --- | --- | --- |
| **Change in FEV1** | **DF** | **X^2^** | **P(>\|Chi\|)** | **Est (95% CI)** | **X^2^** | **P(>\|Chi\|)** | **Est (95% CI)** |
| Age | 1 | 1.347 | 0.246 | 0.486 (-1.141, 2.112) | 4.000 | 0.045* | 1.206 (0.478, 1.934) |
| Gender | 1 | 1.347 | 0.192 | 0.301 (-4.917, 5.52) | 0.059 | 0.809 | 0.66 (-2.745, 4.065) |
| Baseline FEV1 group | 1 | 4.851 | 0.028* | 2.352 (-8.037, 12.742) | 1.068 | 0.301 | -6.682 (-11.819, -1.545) |
| ΔIsc | 1 | 0.197 | 0.657 | 0.136 (-0.015, 0.286) | 7.634 | 0.022* | 0.078 (0.022, 0.134) |
| CFTR modulator | 2 | 6.237 | 0.044* |  | 0.262 | 0.609 |  |
| lum/iva |  |  |  | -21.324 (-41.088, -1.561) |  |  | -0.696 (-8.797, 7.406) |
| tez/iva |  |  |  | 2.352 (-8.037, 12.742) |  |  | -8.297 (-17.579, 0.986) |
| Baseline FEV1 group * ΔIsc | 1 | 3.398 | 0.065^.^ | -0.197 (-0.418, 0.023) | 11.425 | 0.001*** | -0.322 (-0.514, -0.13) |
| CFTR modulator * ΔIsc | 2 | 7.002 | 0.030* |  | 7.885 | 0.019* |  |
| lum/iva |  |  |  | -0.086 (-0.439, 0.267) |  |  | 0.155 (0.039, 0.271) |
| tez/iva |  |  |  | -0.287 (-0.54, -0.033) |  |  | -0.092 (-0.255, 0.07) |

Significance. codes: 0 ‘***’ 0.001 ‘**’ 0.01 ‘*’ 0.05 ‘.’ 0.1

Model 3: Change in SC ~ Age + Gender + CFTR modulator * ΔIsc in differentiated-HNEC cultures

Model 4: Change in SC ~ Age + Gender + ΔIsc in differentiated-HNEC cultures

**C: Analysis of Model 3 and Model 4**

|  |  |  |  |  | **Drug removed** | | |
| --- | --- | --- | --- | --- | --- | --- | --- |
| **Change in SC** | **DF** | **X^2^** | **P(>\|Chi\|)** | **Est (95% CI)** | **X^2^** | **P(>\|Chi\|)** | **Est (95% CI)** |
| Age | 1 | 0.625 | 0.429 | -1.08 (-2.464, 0.303) | 0.625 | 0.429 | 0.051 (-1.36, 1.462) |
| Gender | 1 | 1.906 | 0.167 | 13.035 (4.973, 21.096) | 1.906 | 0.167 | 7.456 (-1.432, 16.344) |
| CFTR modulator | 2 | 196.913 | <0.001*** |  |  |  |  |
| lum/iva |  |  |  | 50.887 (38.155, 63.619) |  |  |  |
| tez/iva |  |  |  | 53.203 (46.443, 59.963) |  |  |  |
| ΔIsc | 1 | 0.595 | 0.441 | -0.092 (-0.22, 0.037) | 7.043 | 0.008** | 0.26 (0.068, 0.452) |
| CFTR modulator  * ΔIsc | 2 | 36.737 | <0.001*** |  |  |  |  |
| lum/iva |  |  |  | 1.27 (0.709, 1.832) |  |  |  |
| tez/iva |  |  |  | -0.245 (-0.404, -0.087) |  |  |  |

Significance. codes: 0 ‘***’ 0.001 ‘**’ 0.01 ‘*’ 0.05 ‘.’ 0.1
