# Supplementary material for: Predictive capacity of paediatric nasal epithelial cells in sequential CFTR modulator therapy
Source: Thorax. 2025 Dec 7;81(6):e223153. doi: 10.1136/thorax-2025-223153 (PMC13217057; doi:10.1136/thorax-2025-223153)
Supplement: online supplemental file 2 [file thorax-81-6-s002.docx]

### **Supplementary file #2**

**Materials and Methods**

**LC-MS to measure CFTR modulator drug levels**

Participants with a blood sample stored in our CF biobank that was collected whilst receiving CFTR modulator treatment were identified. Samples were collected from participants opportunistically during routine collection of blood samples for clinically indicated reasons. Plasma and serum were frozen and stored at -80°C. Liquid chromatography/mass spectroscopy (LC-MS) was used to quantify ivacaftor (IVA), lumacaftor (LUM), and/or tezacaftor (TEZ) in plasma or serum as previously described [1, 2] Plasma or serum were processed with 0.1% formic acid/ACN in a 1:2 dilution ratio. Mixtures were centrifuged (10 minutes, 132,000 rpm). Supernatant were transferred into LC-MS vials. The LC-MS analysis was performed using Shimadzu LC-MS 8050 triple quadrupole mass systems.

***In vivo* clinical response to modulator – FEV1 percent predicted (pp)**

Spirometry was performed by a respiratory scientist in an accredited respiratory laboratory as part of routine CF care [3]. Global Lung Index (GLI) references were used for all spirometry data. The clinical status of the patient was recorded at each time point to identify exacerbation events or intercurrent illnesses, as well as any dose adjustments to modulator treatments. Spirometry results obtained during exacerbations were excluded.


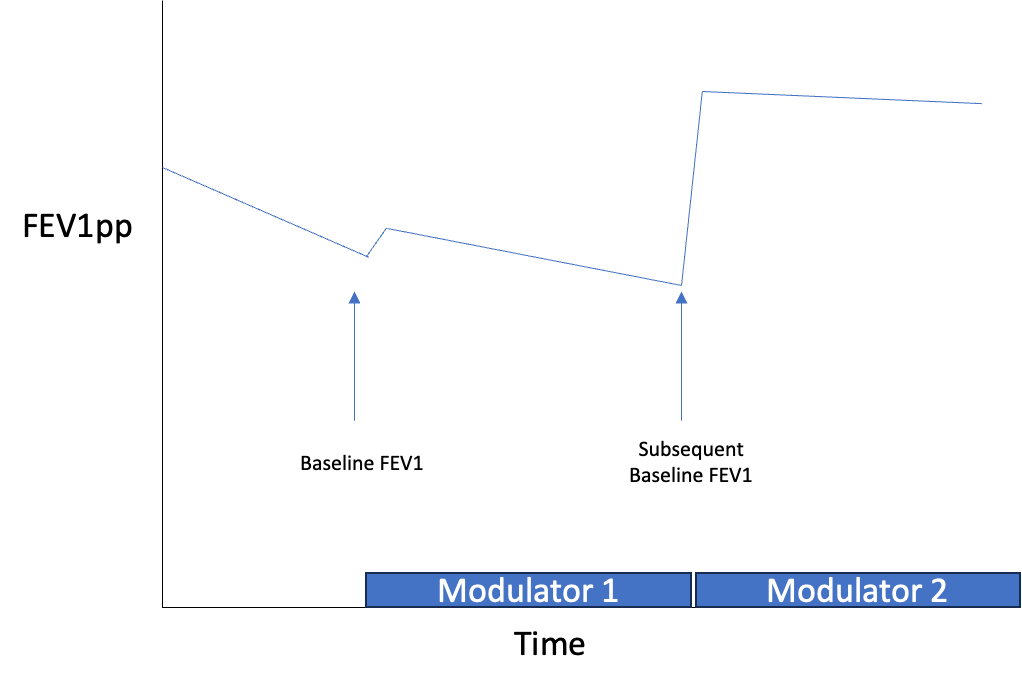
Baseline FEV1pp was defined as the result obtained closest to the introduction of each CFTR modulator treatment to the patient’s treatment plan. Post treatment FEV1pp was defined as the earliest FEV1pp result obtained after at least six weeks of stable CFTR modulator dosing. Six weeks was chosen to reduce the impact of the transient bronchoconstriction side effect associated with initiation of LUM/IVA treatment [4]. Due to the progressive nature of CF lung disease, when participants changed CFTR modulator treatment the FEV1pp response was calculated as the change between the FEV1pp measurement obtained immediately prior to the first dose of the subsequent CFTR modulator (subsequent baseline, **Figure M1**) and the FEV1pp result obtained after at least six weeks of stable subsequent CFTR modulator dosing. The median time to clinical response assessment for the first modulator was 14 weeks (range up to 24 weeks); mean 15.4 weeks. For subsequent modulators, the median time was 9 weeks (range up to 17 weeks); mean 10.3 weeks.

Figure M 1: Schematic of baseline FEV1pp definition

***In vivo* clinical response to modulator – sweat chloride (SC)**

SC testing was performed by an accredited lab (NSW Health pathology) as part of routine clinical care of patients receiving a CFTR modulator at the SCH CF clinic. SC results were obtained from the participant’s medical record. Baseline SC was taken as the measurement prior to any CFTR modulator being taken by the patient. When a SC level immediately pre CFTR modulator treatment was not available, prior results from time of diagnosis were used (n = 2 teenage participants, SC results from infancy used as baseline). Post treatment SC results were obtained after at least 4 weeks of stable CFTR modulator dosing. Post treatment SC results for subsequent CFTR modulator treatments were compared to the baseline results, for the purpose of comparison to the participant’s *in vitro* data.

***CFTR* gene sequencing analysis and drug response relationship**

The DNeasy® Blood & Tissue kit (Qiagen 69506) and the associated protocol was used to extract DNA from participant cells. Whole genome sequencing (WGS) were carried out at Ramaciotti Centre for Genomics (UNSW). DNA libraries were prepared using Illumina DNA prep kit and sequenced in 2x150bp paired-end format on Illumina iseq 100 system. Sequence data were processed using DRAGEN Germline Pipeline (v.4.1.7) for alignment, variant calling and quality control. Reads were mapped to alt-contig masked hg38 reference genome. Analysis was performed using the Gadi system of the National Computational Infrastructure (NCI). The CFTR gene was processed from promoter to the 3’ UTR terminus (GRCh38 coordinates chr7:117476071-117668665). VCF files of each participant for CFTR gene locus were merged using bcftools (V1.12)[5] excluding multiallelic records (-m none) and assuming reference homozygous genotypes (0/0) at invariable sites (-0). Variant consequence and clinical significance were annotated using Ensembl Variant Effect Predictor (V111) [6, 7]. VCF files were converted to GRCh37 coordinates using Ensembl Assembly Converter and phased using ShapeIT2 (v2, r904) [8] with the 1000 Genomes Phase 3 [9] genetic map and default parameters.

**Pharmacogene profiling**

To identify genetic variations influencing drug metabolism and transporter activity, WGS data was analyzed using Stargazer (Version 1.19.2)[10] to detect copy number variations (CNVs), single nucleotide polymorphisms (SNPs), and structural variants in pharmacogenes. Using patient BAM files as input, Stargazer’s –create-gdf-file function was employed to calculate read depth for all pharmacogenes, normalising against RYR1 as the control gene. After generating the GDF (GATK-DepthOfCoverage format) files, patient VCF files was used to call star alleles. The predicted activity levels of enzymes and transporters were categorized based on Stargazer output, focusing on four families; CYP (Cytochrome P450) enzymes, ABC (ATP-binding cassette) transporters, SLC and SLCO (solute carrier) families and UGT (UDP-glucuronosyltransferases). Activity levels were visualized in a heatmap in R using the pheatmap package (Version 1.0.12)[11] with color-coding to indicate variability in activity levels.

**Creation of *in vitro* nasal epithelial cell cultures from patients**

Participants underwent brushing of the inferior nasal turbinates (Endoscan, 33009-SA McFarlane, Ringwood, VIC, Australia) to obtain epithelial cells as previously described [12, 13]. Briefly, collected cells were seeded in a collagen-I precoated flask (Advanced Biomatrix 5015) and co cultured with irradiated NIH/3T3 feeder cells. Human nasal epithelial cells (HNECs) were expanded using a conditional reprogramming culture (CRC) method, supplemented with ROCK (Rho kinase) inhibitor (10 μM) [14]. At 90% confluence, a double trypsin method was used to dissociate the HNECs, which were then cryopreserved at passage 1 in our CF biobank [12].

**Cilia beat frequency measurement**

Functional assessment of differentiated-HNEC cultures was conducted by measuring cilia beat frequency, as described previously [12]. Briefly, mature differentiated-HNEC cultures were imaged using high speed live cell imaging system (Eclipse Ti2-E, Nikon microscope, Andor Zyla 4.2 sCMOS camera, CFI S Plan Fluor ELWD 20×/0.45 objective) in an environmentally controlled chamber (37°C, 85% humidity and 5% CO2). Time-lapse images were acquired and CBF was analysed using a custom-built script in MATLAB (MathWorks, Natick, MA) [12, 15]. Six fields of view (512 x 512 pixels) were acquired from each transwell insert, with the mean of the six images being calculated as the result. A minimum of four filters were measured for each participant at baseline.

**Quantification of *in Vitro* CFTR-mediated ion transport electrophysiology**

CFTR-mediated ion transport was assessed by measurement of short circuit current (I_SC_) in the differentiated-HNEC cultures as described previously [14]. Briefly, differentiated-HNEC cultures were pre-incubated with CFTR correctors (3 µM LUM (VX-809; Selleckchem 1565 [16]), 5 µM TEZ (VX-661; Selleckchem S7059 [17], or a cocktail of 18 µM TEZ and 3 µM elexacaftor (E) (VX-445; Selleckchem S8851) [18] for 48h prior to assessment of ion transport electrophysiology. I_sc_ measurements were taken under voltage-clamp conditions using VCC MC8 Ussing chambers (Physiologic Instruments, San Diego, CA). Data recordings were acquired using Acquire and Analyze (version 2.3) software (Physiologic Instruments, San Diego, CA). Experiments were performed under a basal to apical Cl- concentration gradient, created using 10 mM HEPES buffered Ringer solutions. Basal solution contained (mM): 145 NaCl, 3.3 K_2_HPO_4_, 10 N-2-hydroxyethylpiperazine-N-2-ethane sulfonic acid (HEPES), 10 D-Glucose, 1.2 MgCl_2_, and 1.2 CaCl_2_. Apical solution contained (mM): 145 Na-Gluconate, 3.3 K_2_HPO_4_, 10 HEPES, 10 D-Glucose, 1.2 MgCl_2_, 1.2 CaCl_2_. Ringer solutions were continuously gassed with 95% O_2_-5% CO_2_ and maintained at 37˚C. Following a 30 minute stabilisation period, differentiated-HNEC cultures were treated with pharmacological compounds (in order): 100 µM amiloride (Sigma A7410, apical) to inhibit epithelial sodium channel (ENaC)-mediated sodium (Na^+^) flux, vehicle control 0.01% DMSO or 10 µM IVA (VX-770; Selleckchem S1144; apical) to potentiate cAMP-activated currents, 10 µM forskolin (Sigma F6886, basal) to induce cAMP activation of CFTR, 30 µM CFTR_inh_-172 ( Selleckchem S7139, apical) to inhibit CFTR-specific currents and 100 µM ATP (Sigma A2383, apical) to activate calcium-activated chloride currents (**Figure 5A**).

Wild-type CFTR function values were obtained using the same assay protocol on control nasal epithelial cultures as described in Wong SL et al., 2022 [16]. These values served as the reference for calculating %WT CFTR activity. Functional rescue was calculated using ΔIsc (Forsk + CFTRInh) as the primary measure. Inter-filter variability was greater for ETI (mean SE: 1.67 Ω·cm² or 5.1% WT; range 0.23–4.65) than for LUM/IVA or TEZ/IVA (mean SE: 0.68 Ω·cm² or 2.1% WT; range 0.12–1.99).

**Supplementary results**

**Modulator treatment regimen**

Patients received age-appropriate CFTR modulators as part of their routine CF care. Twelve participants underwent changes in their CFTR regimen during the study period. Four participants transitioned directly from LUM/IVA to ETI, another four from TEZ/IVA to ETI and the remaining four transitioned from LUM/IVA to TEZ/IVA before subsequently transitioning to ETI (**Figure 1A**).

**Heterogenous *in vivo* clinical response (FEV1pp and SC)**

After evaluating the clinical response of all 24 participants to their first CFTR modulator, we evaluated the clinical response of the eight participants who switched their modulator treatment directly to ETI. After treatment with ETI, FEV1pp increased by a mean of 10.00 percentage points (95% CI 5.79 – 14.21, **Figure 2C**), from their subsequent baseline following prior modulator treatment, although the response remained heterogenous between participants (**Figure S2C**). All participants had a significant (> 20mmol/L) decrease in SC compared to their original baseline levels, although the variability in the magnitude of response was large (Mean 55.86 mmol/L, 95% CI; 39.71 – 72.00, **Figure 2D**). Two participants had minimal additional improvement in SC with ETI compared to their first modulator (**Figure S2**).

We also evaluated the clinical response of the four participants who transitioned from LUM/IVA to TEZ/IVA before switching to ETI. Only one participant showed a significant increase in FEV1pp with TEZ/IVA treatment (#18: 5.34 percentage points, **Figure 2E, S2**). However, there were no significant (> 20mmol/L) changes in SC levels compared to baseline for any of the four participants (**Figure 2F, S2**). With ETI treatment, two participants had significant increase in FEV1pp (#5: 19.00 percentage points; #11: 24.47 percentage points, **Figure 2E, S2**). The two participants without significant change in FEV1pp had baseline measurements above 90 (#18 and #20). Three participants who had available SC data (#18, #20 and #5) showed a significant (> 20mmol/L) decrease in SC levels, ranging from a 46 to 59 mmol/L decrease compared to baseline (**Figure 2F, S2**).

**ROC analysis using alternative values for significant clinical response.**

We ran our ROC analysis with lower clinical cut off values to categorise participants as clinical responders or non-responders, The mean change in FEV1pp and SC from the relevant phase III clinical trials [19–21] was used to select cut off values for this additional analysis. This change in cut off value resulted in two tez/iva and two lum/iva participants being considered sweat chloride responders who were previously considered non responders. When applying the lower cut off to participants receiving tez/iva only, the AUC retained its significance with an AUC of 0.875 (95% CI 0.691 – 1.000, p=0.029).

|  | **Original clinical cut offs** | **FEV1pp > 2.5pp increase** | **SC> 10mmol/L**  **(all)** | **SC> 10mmol/L (tez/iva)** |
| --- | --- | --- | --- | --- |
| **FEV1** | 0.62  (0.39 – 0.85) | 0.58  (0.32 – 0.83) |  |  |
| **Sweat Chloride** | **0.88**  **(0.71 – 1.00)** |  | 0.79  (0.45 – 1.00) | **0.88**  **(0.69 – 1.00)** |
| **Both** | **0.77**  **(0.53 – 1.00)** |  | 0.67  (0.36 – 0.99) | 0.75  (0.48 – 1.00) |
| **FEV1 40-90** | **1.00**  **(1.00-1.00)** | **0.88**  **(0.67 – 1.00)** |  |  |
| **Both** | **1.00**  **(1.00-1.00)** |  | 0.67  (0.22 – 1.00) | 0.81  (0.47 – 1.00) |
| **FEV1 > 90** | 0.72  (0.42 – 1.00) | 0.67  (0.33 – 1.00) |  |  |
| **Both** | 0.50  (0.07 – 0.93) |  | 0.55  (0.13 – 0.97) | 0.55  (0.13 – 0.97) |

References

1. Schneider EK, Reyes-Ortega F, Li J, et al. Optimized LC-MS/MS Method for the High-throughput Analysis of Clinical Samples of Ivacaftor, Its Major Metabolites, and Lumacaftor in Biological Fluids of Cystic Fibrosis Patients. *J Vis Exp; 2017*: 56084.
2. Reyes-Ortega F, Qiu F, Schneider-Futschik EK. Multiple Reaction Monitoring Mass Spectrometry for the Drug Monitoring of Ivacaftor, Tezacaftor, and Elexacaftor Treatment Response in Cystic Fibrosis: A High-Throughput Method. *ACS Pharmacol Transl Sci;* 2020; 3: 987–996.
3. Castellani C, Duff AJA, Bell SC, et al. ECFS best practice guidelines: the 2018 revision. *Journal of Cystic Fibrosis Elsevier;* 2018; 17: 153–178.
4. Wainwright CE, Elborn JS, Ramsey BW, et al. Lumacaftor–Ivacaftor in Patients with Cystic Fibrosis Homozygous for Phe508del CFTR. *New England Journal of Medicine*; 2015; 373: 220–231.
5. Danecek P, Bonfield JK, Liddle J, et al. Twelve years of SAMtools and BCFtools. *Gigascience*; 2021; 10: 1–4.
6. McLaren W, Gil L, Hunt SE, et al. The Ensembl Variant Effect Predictor. *Genome Biol*; 2016; 17: 1–14.
7. Martin FJ, Amode MR, Aneja A, et al. Ensembl 2023. *Nucleic Acids Res*; 2023; 51: D933–D941.
8. Delaneau O, Marchini J, Zagury JF. A linear complexity phasing method for thousands of genomes. *Nature Methods* 2011 9:179–181.
9. Auton A, Abecasis GR, Altshuler DM, et al. A global reference for human genetic variation. *Nature;* 2015; 526: 68–74.
10. Lee S been, Wheeler MM, Thummel KE, *et al.* Calling Star Alleles With Stargazer in 28 Pharmacogenes With Whole Genome Sequences. *Clin Pharmacol Ther*; 2019;106: 1328–1337.
11. GitHub - raivokolde/pheatmap: Pretty heatmaps [Internet]. [cited 2024 Nov 30]. Available from: https://github.com/raivokolde/pheatmap.
12. Allan KM, Wong SL, Fawcett LK, et al. Collection, Expansion, and Differentiation of Primary Human Nasal Epithelial Cell Models for Quantification of Cilia Beat Frequency. *JoVE*; 2021; e63090.
13. Fawcett LK, Turgutoglu N, Allan KM, *et al.* Comparing Cytology Brushes for Optimal Human Nasal Epithelial Cell Collection: Implications for Airway Disease Diagnosis and Research. *J Pers Med*; 2023;13.
14. Awatade NT, Wong SL, Capraro A, et al. Significant functional differences in differentiated Conditionally Reprogrammed (CRC)- and Feeder-free Dual SMAD inhibited-expanded human nasal epithelial cells. *Journal of Cystic Fibrosis Elsevier*; 2021; 20: 364–371.
15. Wong SL, Awatade NT, Astore MA, et al. Molecular Dynamics and Theratyping in Airway and Gut Organoids Reveal R352Q-CFTR Conductance Defect. *Am J Respir Cell Mol Biol;* 2022; 67: 99–111.
16. Pranke IM, Hatton A, Simonin J, et al. Correction of CFTR function in nasal epithelial cells from cystic fibrosis patients predicts improvement of respiratory function by CFTR modulators. *Scientific Reports* 2017; 7: 1–11.
17. Allan KM, Astore MA, Fawcett LK, et al. S945L-CFTR molecular dynamics, functional characterization and tezacaftor/ivacaftor efficacy in vivo and in vitro in matched pediatric patient-derived cell models. *Front Pediatr*; 2022; 10: 1062766.
18. Keating D, Marigowda G, Burr L, et al. VX-445-Tezacaftor-Ivacaftor in Patients with Cystic Fibrosis and One or Two Phe508del Alleles. *N Engl J Med*; 2018;379: 1612–1620.
19. Wainwright CE, Elborn JS, Ramsey BW, *et al.* Lumacaftor–Ivacaftor in Patients with Cystic Fibrosis Homozygous for Phe508del CFTR . *New England Journal of Medicine*; 2015; 373: 220–231.
20. Milla CE, Ratjen F, Marigowda G, *et al.* Lumacaftor/Ivacaftor in patients aged 6-11 years with cystic fibrosis and homozygous for F508del-CFTR. *Am J Respir Crit Care Med*; 2017; 195: 912–920.
21. Taylor-Cousar JL, Munck A, McKone EF, *et al.* Tezacaftor–Ivacaftor in Patients with Cystic Fibrosis Homozygous for Phe508del. *New England Journal of Medicine*; 2017; 377: 2013–2023.
