# Supplementary figures and images for: Predictive capacity of paediatric nasal epithelial cells in sequential CFTR modulator therapy
Source: Thorax. 2025 Dec 7;81(6):e223153. doi: 10.1136/thorax-2025-223153 (PMC13217057; doi:10.1136/thorax-2025-223153)

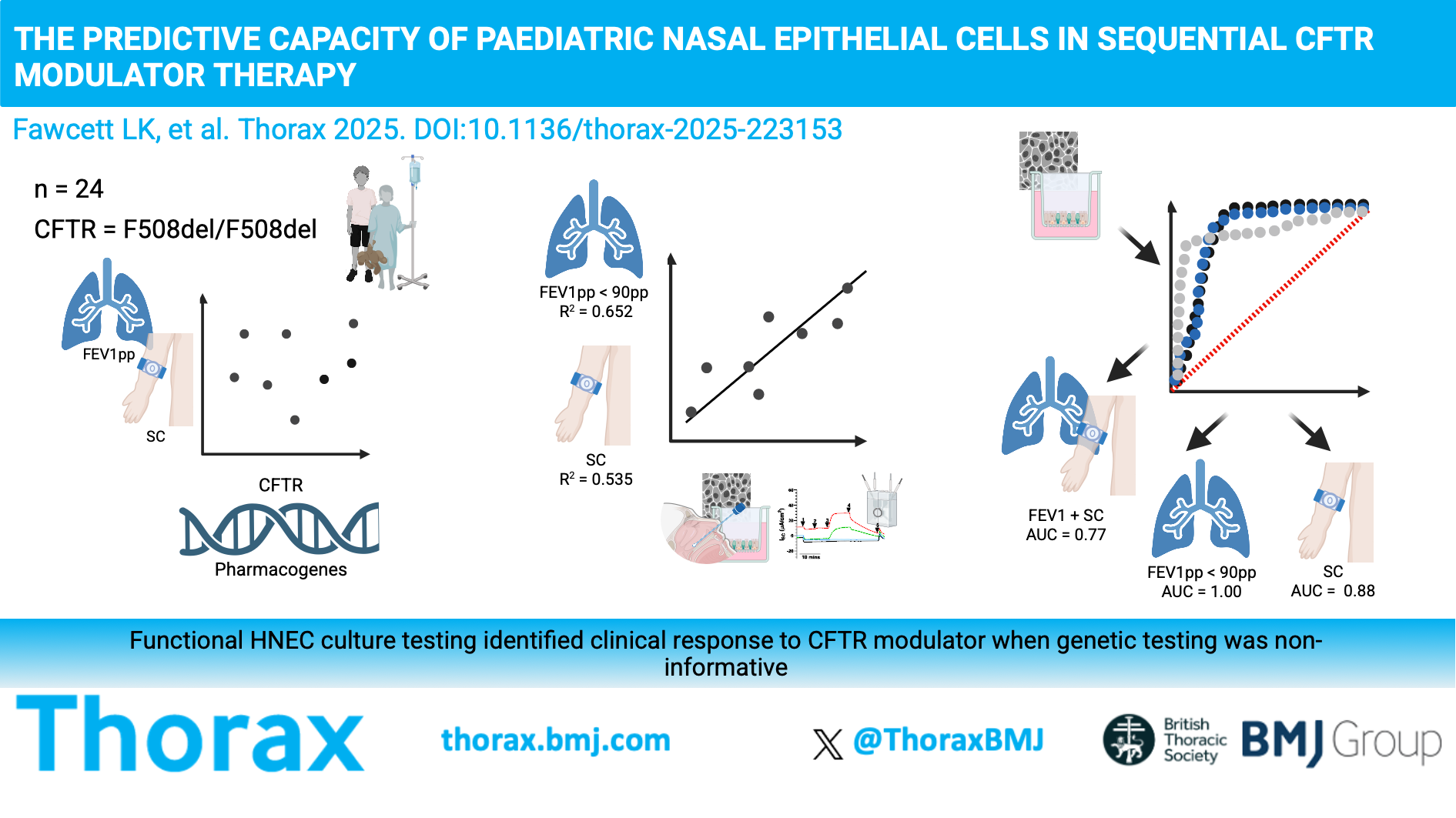

Supplement: online supplemental file 3 [file thorax-81-6-s003.png]
